# Intrathecal delivery of recombinant AAV1 encoding hepatocyte growth factor improves motor functions and protects neuromuscular system in the nerve crush and SOD1-G93A transgenic mouse models

**DOI:** 10.1186/s40478-019-0737-z

**Published:** 2019-06-12

**Authors:** Sang Hwan Lee, Subin Kim, Nayeon Lee, Junghun Lee, Seung Shin Yu, Jin Hong Kim, Sunyoung Kim

**Affiliations:** 1 R&D Center for Innovative Medicines, Helixmith Co., Ltd., Building 203, Gwanak-Gu, Seoul, 08826 Korea; 2 School of Biological Sciences, Seoul National University, Gwanak-Gu, Seoul, 08826 Korea

**Keywords:** Amyotrophic lateral sclerosis (ALS), Adeno-associated virus (AAV), Hepatocyte growth factor (HGF), Corticospinal motor neuron (CSMN), Oxidative stress

## Abstract

Amyotrophic lateral sclerosis (ALS) is a fatal neuromuscular disease resulting from motor neuron degeneration that causes muscle weakness, paralysis, and eventually respiratory failure. We investigated whether recombinant adeno-associated virus encoding human hepatocyte growth factor (rAAV-HGF) could generate beneficial effects in two mouse models with neuromuscular problems when intrathecally delivered to the subarachnoid space. We chose AAV serotype 1 (rAAV1) based on the expression levels and distribution of HGF protein in the lumbar spinal cord (LSC). After a single intrathecal (IT) injection of rAAV1-HGF, the protein level of HGF in the LSC peaked on day 14 and thereafter gradually decreased over the next 14 weeks. rAAV1-HGF was initially tested in the mouse nerve crush model. IT injection of rAAV1-HGF improved mouse hindlimb strength and rotarod performance, while histological analyses showed that the length of regenerated axons was increased and the structure of the neuromuscular junction (NMJ) was restored. rAAV1-HGF was also evaluated in the SOD1-G93A transgenic (TG) mouse model. Again, rAAV1-HGF not only improved motor performance but also increased the survival rate. Moreover, the number and diameter of spinal motor neurons (SMNs) were increased, and the shape of the NMJs restored. Data from in vitro motor cortical culture experiments indicated that treatment with recombinant HGF protein (rHGF) increased the axon length of corticospinal motor neurons (CSMNs). When cultures were treated with an ERK inhibitor, the effects of HGF on axon elongation, protein aggregation, and oxidative stress were suppressed, indicating that ERK phosphorylation played an important role(s). Taken together, our results suggested that HGF might play an important role(s) in delaying disease progression in the SOD1-G93A TG mouse model by reducing oxidative stress through the control of ERK phosphorylation.

**Electronic supplementary material:**

The online version of this article (10.1186/s40478-019-0737-z) contains supplementary material, which is available to authorized users.

## Introduction

ALS is a neurodegenerative disease, in which the motor neurons of the central nervous system degenerate progressively [13, 23, 49]. A variety of chemicals, recombinant proteins, and gene therapies have been explored to develop treatment methods for ALS, but to date, only two small molecules, riluzole and edaravone, have been approved by the FDA [3, 5, 39]. Their therapeutic effects are relatively low: riluzole delays the time to tracheostomy by 2 to 3 months, and edaravone improves the ALSFRS-R score by 33%. There is still a high unmet need for a medical solution to this fatal disease.

In this study, we investigated whether HGF could be used to treat ALS in the context of AAV-based gene therapy. Since its discovery in 1984, HGF has been shown to contain multiple bioactivities, promoting cell proliferation, morphogenesis, anti-fibrosis, anti-inflammation, and angiogenesis, among others [17, 28, 31, 32, 40, 52, 57]. When HGF binds to the Met receptor, a variety of intracellular signaling pathways are activated, involving PI3K, JNK, p38, and/or ERK, triggering different biological reactions depending on the cell type [36, 37, 50]. HGF is also a neurotrophic factor in that it can induce the development, proliferation, and differentiation of neuronal cells [19, 30, 34, 41, 45, 46]. It has also been reported that the HGF-Met complex was abnormally sequestered in fALS patients [20]. In the SOD1-G93A TG mouse model, disease progression has been delayed when HGF or Met expression was additionally introduced by generating double TG mice, or when rHGF was intrathecally delivered, indicating that HGF might play an important role(s) in ALS [10, 16, 48].

HGF-expressing plasmid DNA was used in the phase I trial for ALS [47]. A single intramuscular administration of this plasmid DNA in 18 ALS patients has been proven to be safe, with no significant adverse effects. According to the ALSFRS-R scores, the conditions of patients appeared to stabilize or even improve in 50% of patients—albeit temporarily—during the first 2 to 3 months following injection. The same plasmid was also used for diabetic peripheral neuropathy and critical limb ischemia, and generated highly positive results with virtually no drug-related adverse effects, indicating the possibility of employing plasmid DNA expressing HGF for a broad range of neuromuscular or neuroischemic diseases [1, 11, 14, 21, 22].

This study employed AAV, as an alternative method of delivering the HGF gene [7, 59]. AAV can drive relatively long-term expression in vivo and deliver the transgene to a variety of cell types, including non-dividing cells through capsid pseudotyping [7]. We found that among 4 serotypes tested, serotype 1 (rAAV1) most effectively delivered the gene to the ventral horn of the LSC when introduced to the subarachnoid space by IT delivery. When evaluated in two mouse models—the sciatic nerve crush and SOD1-G93A TG mouse models—rAAV1-HGF ameliorated deficits in motor functions as measured by several behavioral tests and regenerated impaired NMJ structure [53]. Consistent with these observations, HGF was found to increase the axon lengths of CSMNs in vitro. Data from experiments involving specific chemical inhibitors revealed that phosphorylation of ERK is an important player for HGF-generated effects. Our results suggested that the HGF gene may be a good starting point for developing therapeutic agents for ALS and that rAAV vector in particular might be a useful gene delivery vehicle.

## Materials & methods

### Animals

C57BL/6 mice were purchased from Orient Bio Inc. (Gyeonggi-do, Korea). B6SJL-TG(SOD1-G93A)1Gur/J mice (MGI ID: 2183719) were purchased from The Jackson Laboratory [12, 51, 55]. Mice were fed ad libitum and entrained to a standard light-dark cycle. All experimental protocols adhered to the regulations of the Seoul National University Institutional Animal Care and Use Committee (IACUC).

### Adeno-associated virus

In this study, a gDNA-cDNA-hybrid sequence that could express HGF723 (dHGF) and HGF728 (cHGF) was generated using human genomic DNA as previously described [6, 38]. This hybrid sequence has cDNA sequences of 18 exons of the HGF gene, containing a part of intron 4 between exon 4 and exon 5. Since the length of intron 4 is too long to produce viral vectors, 4329 base pairs of intron 4 were deleted using site-directed mutagenesis by polymerase chain reaction (PCR). This chimeric sequence was then inserted into the ITR (inverted terminal repeat) region of pAAV-MCS, a plasmid containing multiple cloning sites purchased from Agilent Technologies, to generate pAAV-HGF. To produce rAAV2-HGF, equal amounts of three plasmids (*rep*/*cap*-expressing plasmid*,* pAAV-HGF, and helper plasmid) were co-transfected into 1 × 10^6^ HEK293T cells using AAV Helper-Free System (Agilent Technologies). 1.23 × 10^8^ GC (genome copies) of rAAV2-HGF were then transduced into 1.6 × 10^5^ C2C12 cells. Forty-eight hours later, the supernatant was collected followed by ELISA specific to human HGF (hHGF). At the same time, total RNAs were isolated and subjected to RT-PCR using Expand High Fidelity PCR System (Sigma). Primers used for PCR were as follows; 5′-CAAATGTCAGCCCTGGAGTTCCATGA-3′ (forward); 5′-CTGGATTGCTTGTGAAACACCAGGGT-3′ (reverse). PCR products were then run on NuPAGE 4–12% Bis-Tris Protein Gels (Thermofisher). rAAV2-MCS lacking the HGF sequence (rAAV2-C) was used as a negative control. For animal studies, rAAV (serotype 1, 2, 5, and 6) with a higher titer (1 × 10^12^ GC/ml) was produced by a contract manufacturing company called CdmoGen (Chungbuk, Korea).

### Sciatic nerve crush

The sciatic nerves of C57BL/6 mice were exposed, and nerve crush injury was induced with fine hemostatic forceps (FST), as described previously [6, 24]. Mice were exposed to isoflurane at 4–5% until anesthesia was induced, with concentration reduced to 1.5% during surgery. Behavioral tests were performed once a week for four weeks. For histological analysis, the sciatic nerves and tibialis anterior (TA) muscles were collected five days after surgery.

### Behavioral tests

The motor function of mice was evaluated using the rotarod test, wire hanging test, and grip strength test. For the rotarod test, the latency to fall from the rotating rod was recorded. The speed of the rod was accelerated from 4 rpm to 40 rpm for 5 min, as described previously [15, 25, 44]. For the wire hanging test, mice were placed upside down under wire mesh. The latency to fall was recorded. The maximum latency was set to 1 min. Grip strengths of the forelimb and hindlimb were measured with a grip strength meter (Nidec-Shimpo). Mice were pre-trained for a week for each behavioral test. Each test was performed three times and values were averaged.

### Primary motor cortical cultures

Cerebral motor cortices of mice were isolated, and cells were incubated as described previously [18]. Six-well cell culture plates (SPL) pre-coated with 0.1 mg/ml of PDL (Sigma) were incubated overnight at 37 °C, followed by two washings with distilled water (Sigma). Motor cortices of non-TG or TG mice at P3 were collected using fine forceps (FST) into dissociation solution (DS) containing magnesium chloride (Sigma), Hepes (Gibco), sodium sulfate (Sigma), potassium sulfate (Sigma), kyneuric acid (Sigma), glucose (Sigma), APV (Sigma), penicillin/streptomycin (P/S) (Gibco), and B27 (Gibco). Cells were then dissociated in a papain solution (Worthington Biochem) for 15 min, and incubated in inhibitor solution containing ovomucoid (Sigma) for 1 min. After being washed with Opti-MEM (Gibco) solution containing APV (Sigma) and B27, 3.2 × 10^5^ cells were seeded with serum-free media (SFM) containing BSA, L-glutamine (Sigma), P/S, glucose, and B27 in neurobasal media (Gibco). Cells were then treated with inhibitors for Met (PHA665752), ERK (U0126), PI3K (LY294002), p38 (SB203580), and JNK (SP600125). Thirty minutes later, cells were cultured in the presence of 100 ng/ml of rHGF (R&D Systems). 3 days later, cells were subjected to immunocytochemistry (ICC) assay.

### Western blot

Total proteins were isolated using RIPA Buffer (Cell Signaling Technology) and Protease/Phosphatase Inhibitor Cocktail (Cell Signaling Technology), followed by polyacrylamide gel electrophoresis on NuPAGE 4–12% Bis-Tris Protein Gels (Thermofisher) using 10–20 μg of protein. Gels were transferred to the PVDF membrane (GE Healthcare) and blocked with 0.1% TBST solution containing 5% skim milk (Difco) for 1 h. Membranes were incubated with 0.1% TBST solution containing 5% BSA and primary antibodies for 1 h, then treated with 0.1% TBST solution containing 5% skim milk for 1 h. HRP conjugated anti-rabbit or anti-mouse IgG (Sigma) was used as a secondary antibody. Membranes were incubated with Immobilin Western Chemiluminescent HRP Substrate (Millipore) for 1 min and developed on X-ray film (AGFA).

### Immunohistochemistry (IHC)

The sciatic nerves, LSCs, and TA muscles were fixed in 4% paraformaldehyde (Sigma) at 4 °C overnight. After being washed three times with 0.1 M PBS, the tissues were immersed in 0.1 M PBS containing 30% sucrose (Sigma) at 4 °C overnight, followed by cryopreservation in OCT compound (Sakura Tissue Tek). Samples were then cryosectioned using Cryostat (Leica). After 1 h incubation in blocking solution containing 2% BSA, 5% normal donkey serum (Jackson ImmunoResearch), and 0.1% Triton X-100 (Samchun), samples were treated with blocking solution containing primary antibodies for 1 h, and then with blocking solution containing secondary antibodies for 1 h. IgG Alexa Fluor (Invitrogen) was used as a secondary antibody. After mounting tissue sections on microscope slides (Fisher Scientific) with DAPI (Vectashield), immunofluorescence was observed using LSM 700 confocal laser scanning microscopy (Carl Zeiss).

### Immunocytochemistry (ICC)

Cells were fixed in 4% paraformaldehyde for 10 min at room temperature. After being washed three times with PBS, cells were immersed in 0.1 M PBS containing 2% Triton X-100 for 5 min at 4 °C, followed by one hour incubation in blocking solution with 2% normal donkey serum and 1% BSA. Cells were then treated with blocking solution containing primary antibodies for 1 h, followed by another 1-h incubation in blocking solution with secondary antibodies. IgG Alexa Fluor was used as a secondary antibody. After mounting cells on microscope slides with DAPI, immunofluorescence was observed using LSM 700 confocal laser scanning microscopy. The axon length of CSMNs was measured using Fiji software (NIH).

### Quantitative real time PCR (q-RTPCR)

The transgene copy number of SOD1-G93A TG mice was measured using q-RTPCR, as described previously [2]. For transgene (hSOD1), the primers used for PCR were as follows; 5′-CATCAGCCCTAATCCATCTGA-3′ (forward); 5′-CGCGACTAACAATCAAAGTGA-3′ (reverse). For reference gene (IL2), the primers used for PCR were as follows; 5′-CTAGGCCACAGAATTGAAAGATCT-3′ (forward); 5′-GTAGGTGGAAATTCTAGCATCATCC-3′ (reverse). After 40 cycles of qPCR using TB Green (Takara), the difference of threshold cycle (ΔCT) was calculated, and mice with values between 6.6 and 7.2 were used.

### Cell viability assay

1.6 × 10^5^ cells were seeded on PDL-coated 48-well cell culture plates (SPL) and incubated for 3 days. After being washed two times with warm PBS, cells were incubated with SFM containing 10% WST1 (AbFrontier) for 1 h at 37 °C. Absorbance was measured at 440 nm using a Magellan microplate reader (Tecan).

### Enzyme-linked immunosorbent assay (ELISA)

To measure the HGF protein level in vitro*,* 1.6 × 10^5^ C2C12 cells were transduced with 5 × 10^13^ GC of rAAV2-HGF. Forty-eight hours later, supernatants were collected followed by ELISA specific to human HGF (R&D Systems). To measure the in vivo expression of HGF, C57BL/6 P60 mice were intrathecally injected with 5 × 10^9^ GC of rAAV1-HGF. The LSCs were collected 1, 2, 4, 8, 12, and 16 weeks after injection. Total proteins were then extracted using RIPA Buffer and Protease/Phosphatase Inhibitor Cocktail, followed by ELISA for hHGF. Absorbance was measured at 450 nm, and wavelength correction was made at 540 nm using a Magellan microplate reader.

### Reactive oxygen species (ROS) detection assay

6.4 × 10^4^ cells were seeded on PDL-coated 96-well cell culture plates (Nunc) and incubated for 3 days. ROS levels were measured using a ROS Detection Assay Kit (Abcam). Cells were incubated with detection solution for 1 h at 37 °C. Pyocianin was used as a ROS inducer, while N-acetyl-L-cysteine was used as a ROS scavenger. Absorbance was measured using a Magellan microplate reader.

### Microarray assay

Motor cortical cells were treated with 100 ng/ml of rHGF, and total RNAs were isolated followed by Microarray analysis using Affymetrix Genechip (Thermofisher). After data extraction, RMA (robust multi-array average) normalization was performed, followed by DEG (differentially expressed gene) analysis. Out of selected DEGs, functional annotation was performed based on GO (gene ontologies) hierarchy and KEGG/BioCarta pathways. Selected genes were clustered according to the classification from the previous report [49].

### Statistical analysis

All values are presented as mean ± standard error mean (SEM). Differences between two values were analyzed by Student’s t-tests. Differences between three or more values were analyzed by one-way ANOVA followed by Tukey’s post-hoc test. For values containing temporal factors, two-way ANOVA was performed followed by Tukey or Sidak’s post-hoc test.

## Results

### Construction of rAAV vectors expressing human HGF

In this study, we tested whether an HGF-expressing rAAV vector could facilitate the regeneration of motor neurons and alleviate disease progression of ALS when delivered to the subarachnoid space through IT injection. AAV used in this study was designed to express two isoforms of human HGF—HGF723 (or dHGF) and HGF728 (or cHGF)—as in the case of our bodies [6]. In order to simultaneously express two isoforms, we used the gDNA-cDNA-hybrid sequence similar to that described by Cho et al. (Additional file 1: Figure S1a). This chimeric sequence was inserted into pAAV-MCS containing multiple cloning sites, resulting in pAAV-HGF.

To produce rAAV vectors, equal amounts of three plasmids (pAAV-HGF, *rep/cap*-expressing plasmid, and helper plasmid) were co-transfected into 1 × 10^6^ HEK293T cells, initially producing AAV serotype 2 [4]. Seventy-two hours later, the supernatant was collected and viral titer was determined as described previously [29]. To determine whether rAAV vectors could indeed produce RNAs of two HGF isoforms, 1.6 × 10^5^ C2C12 cells were transduced with 1.23 × 10^8^ GC of rAAV, and total RNAs were isolated 48 h later followed by RT-PCR. A control virus—rAAV2-C lacking the HGF sequence—did not produce any HGF RNA, whereas two HGF RNA species, each for HGF723 and HGF728, were readily detectable in cells transduced with rAAV2-HGF **(**Additional file 1: Figure S1b**)**.

To test whether HGF proteins were indeed produced from rAAV vectors, 8 × 10^4^ C2C12 cells were transduced with 5 × 10^13^ GC of rAAV, and total proteins were isolated 48 h later followed by ELISA specific to human HGF proteins. rAAV2-HGF produced 159.13 ± 23.92 ng/mg of HGF, while no HGF was detectable in cells transduced with a control vector. (Additional file 1: Figure S1c).

### Comparison of four AAV serotypes

**Fig. 1:**
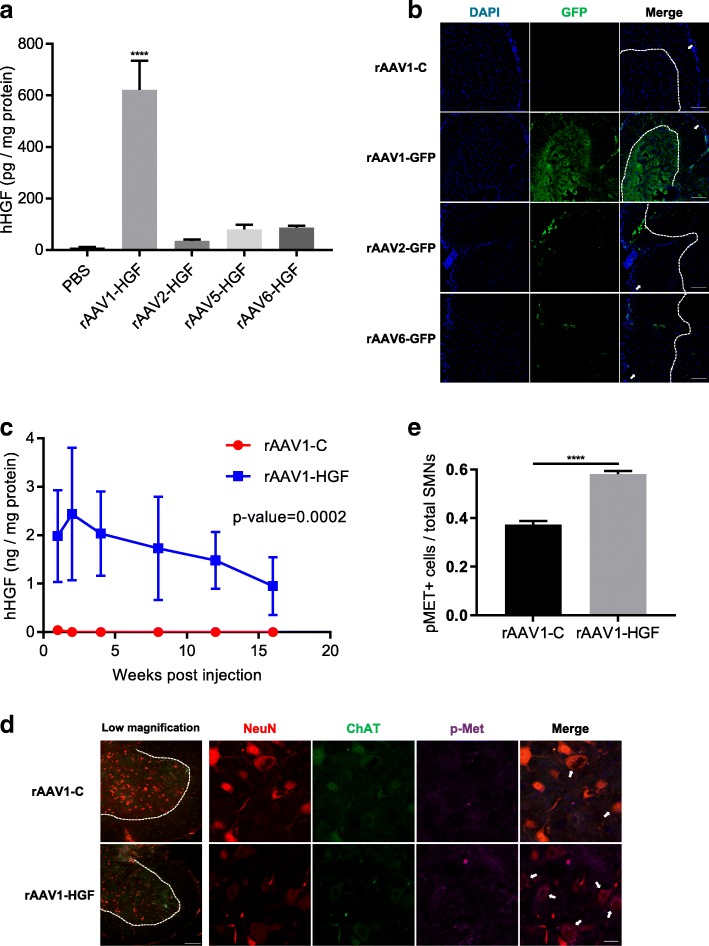
IT administration of rAAV1-HGF promoted functional recovery in the sciatic nerve crush mouse model. **a** 4.12 × 10^8^ GC of rAAV1, 2, 5, or 6-HGF were intrathecally injected into C57BL/6 mice at postnatal day 60 (P60). The LSCs were collected and total proteins were isolated 4 weeks after injection, followed by ELISA specific to human HGF (hHGF). For statistical analysis, one-way ANOVA was performed, followed by Tukey’s post-hoc test. ^****^p < 0.0001 for rAAV1 vs. other serotypes. **b** 1.4 × 10^9^ GC of four serotypes were intrathecally injected into 2-month-old C57BL/6 mice. The LSCs were collected 4 weeks after injection. Tissues were fixed, followed by IHC assay using an antibody specific to GFP (green). The boundary between white and grey matter is distinguished by dotted lines, and white matter is indicated by white arrows. **c** 5 × 10^9^ GC of rAAV1-HGF were intrathecally injected into 2-month-old C57BL/6 mice. The LSCs were collected 1, 2, 4, 8, 12, and 16 weeks after IT injection and subjected to ELISA for hHGF. For statistical analysis, two-way ANOVA was performed, followed by Sidak’s post-hoc test. The *p*-value between the two groups was 0.0002. **d** C57BL/6 mice at P60 were intrathecally injected with 5 × 10^9^ GC of rAAV1-C or rAAV1-HGF. One week later, LSCs were collected and subjected to IHC assay. Antibodies specific to ChAT (green) and NeuN (red) were used to label SMNs, together with those for p-Met (magenta). In low magnification panels, the boundary between white and grey matter is distinguished by dotted lines. In merge panels, p-Met-expressing SMNs are indicated by white arrows. **e** The proportion of SMNs expressing p-Met per total SMNs was measured and represented as a bar graph. For statistical analysis, Student’s t-test was performed. ^****^p < 0.0001. In bar graphs, values are represented as mean ± SEM. Scale bar: **b** = 100 μm, **d =** 100 μm for low magnification panels and 20 μm for the others

Different subtypes of AAV have been shown to possess different tropism. To select an appropriate AAV serotype that could be used for gene transfer to the spinal cord, four serotypes of AAV (rAAV-1, − 2, − 5, and − 6) were prepared as described above, and 4.12 × 10^8^ GC of each AAV were injected into the LSC. Four weeks later, total proteins were extracted from the LSC, and the expression level of HGF was compared using ELISA. As summarized in Fig. 1a, mice injected with rAAV1-HGF produced the highest level of HGF protein (621.89 ± 112.98 pg/mg), and other serotypes generated 7- to 17-fold lower amounts, 86.25 ± 7.59 pg/mg for rAAV6, 35.54 ± 4.87 pg/mg for rAAV2, and 80.15 ± 17.94 pg/mg for rAAV5 **(**Fig. 1a**)**.

To determine the tissue distribution of transgene expression from different AAV vectors inside the LSC, different subtypes of AAV expressing GFP were used because it was technically difficult to analyze human HGF by IHC. Four weeks after IT injection of AAV vectors, tissue samples were taken and GFP-expressing cells were measured by IHC. rAAV1-GFP transduced the largest area of the ventral horn, while rAAV2 or rAAV6 expressed GFP primarily on the surface of white matter **(**Fig. 1b**)**. Taken together, AAV1 appeared to be the most effective serotype for IT gene delivery, and thus was used for further experiments.

### Kinetics of HGF expression from rAAV1-HGF

To determine how the HGF expression level changed over time, 5 × 10^9^ GC of rAAV1-HGF were injected into the LSC of 2-month-old C57BL/6 mice, and total proteins were isolated, followed by ELISA. One week after injection, 1.98 ± 0.95 ng/mg of hHGF were detectable, and the level was the highest after two weeks (2.44 ± 1.37 ng/mg), thereafter gradually decreasing over time until 16 weeks after injection (0.95 ± 0.58 ng/mg) **(**Fig. 1c**)**. A control virus lacking the HGF sequence did not produce any HGF protein. While hHGF was not detectable in the motor cortex and serum, 170.17 ± 155.95 pg/mg were observed in the TA (Additional file 1: Figure S1d).

**Fig. 2:**
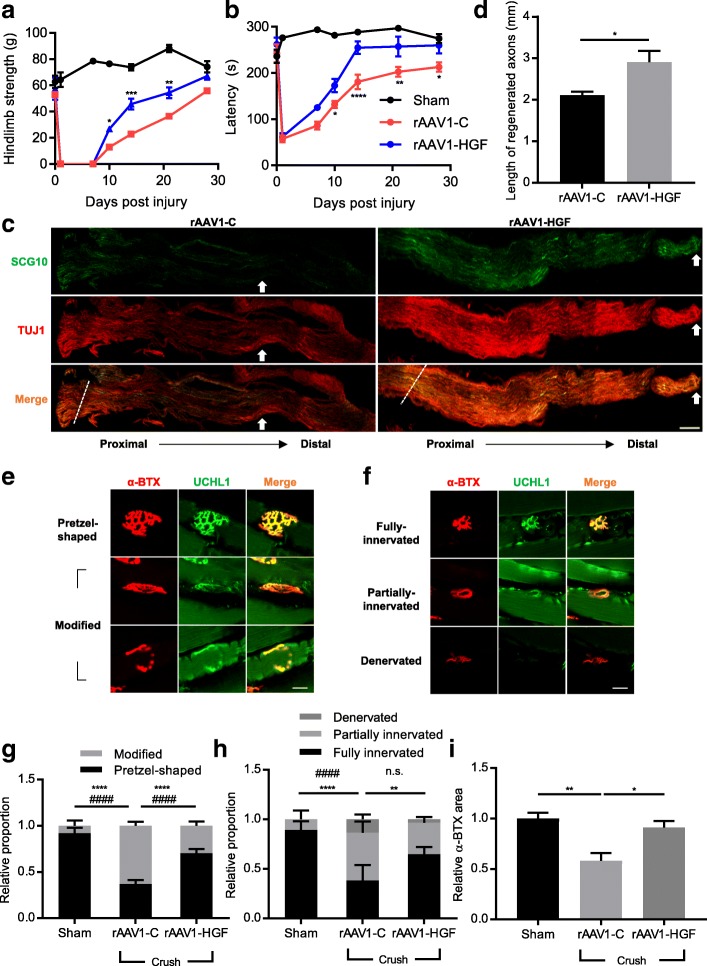
IT delivery of rAAV1-HGF facilitated the regeneration of the sciatic nerves and TA muscles after sciatic nerve crush. **a-h** Using 2-month-old C57BL/6 mice, sciatic nerve crush was induced, and 5 × 10^11^ GC of rAAV1-C or rAAV1-HGF were intrathecally injected into the LSC. Behavioral tests were performed once a week for 28 days (**a-b**). For IHC assay, the sciatic nerves and TA muscles were collected 5 days after nerve crush (**c-f**). **a** Hindlimb strength was measured 1, 7, 10, 14, 21, and 28 days after nerve crush. Value at day 0 was measured before nerve crush. **b** Mean latency to fall from the rotarod was measured 1, 7, 10, 14, 21, and 27 days after nerve crush. Value at day 0 was measured before nerve crush. In Fig. 2a-b, two-way ANOVA was performed, followed by Tukey’s post-hoc test. **c** Representative images of the sciatic nerves from mice treated with rAAV1-C or rAAV1-HGF after nerve crush. Antibodies specific to TUJ1 and SCG10 were used as markers for neurons and regenerating axons, respectively. Crush sites are indicated by dotted lines, and the tip of SCG10 signals is indicated by white arrows. Proximodistal direction is indicated by dotted lines. **d** After sciatic nerve crush, lengths of regenerated nerves were measured using Fiji software and represented as a bar graph. For statistical analysis, Student’s t-test was performed. **e-f** An antibody specific to UCHL1 was used as a marker for presynaptic terminals, whereas that of α-bungarotoxin (α-BTX) was used for postsynaptic end plates [9]. The shapes of NMJs were analyzed to determine whether they were pretzel-shaped or distorted (**e**). The integrity of NMJs was determined by measuring to what degree presynaptic terminals merged with postsynaptic end plates (**f**). **g-i** After sciatic nerve crush, the shape (**g**) and integrity (**h**) of NMJs and average α-BTX area (**i**) were measured. For graphs, values are represented as mean ± SEM. In Fig. 2g-h, two-way ANOVA was performed, followed by Tukey’s post-hoc test. In Fig. 2g, ^****^p < 0.0001 for modified NMJ, ^####^*p* < 0.0001 for pretzel-shaped **NMJ. In** Fig. 2h**,** ^**^p < 0.01 and ^****^p < 0.0001 for fully innervated NMJ, ^####^*p* < 0.0001 and *n.s.* > 0.05 partially innervated NMJ. In Fig. 2i, one-way ANOVA was performed, followed by Tukey’s post-hoc test. For the remaining bar graphs, ^*^p < 0.05, ^**^p < 0.005, ^***^p < 0.001, ^****^p < 0.0001. Scale bars: **c** = 200 μm; **d-e** = 20 μm

To investigate whether the HGF proteins expressed from rAAV1-HGF could increase the phosphorylation of Met, a receptor for HGF, 5 × 10^9^ GC of rAAV1-HGF were intrathecally injected into C57BL/6 mice at P60, Met phosphorylation levels were measured 7 days later using IHC. As shown in Fig. 1d, SMNs were labeled with NeuN (red) and ChAT (green). In mice injected with rAAV1-C, 37.32 ± 0.02% of SMNs were co-stained with p-Met. The number of p-Met-positive SMNs was increased to 58.09 ± 0.01% in the rAAV1-HGF group **(**Fig. 2e**)**. These results indicated that HGF proteins expressed from rAAV1-HGF could indeed augment the phosphorylation of Met.

### Effects of rAAV1-HGF on the sciatic nerve crush model

We first used the sciatic nerve crush mouse model to quickly check the effects of intrathecally delivered rAAV1-HGF on nerve damage in general [53]. Crush injury was introduced in 2-month-old C57BL/6 mice, and 5 × 10^9^ GC of virus were intrathecally injected immediately. As shown in Fig. 2a-b, rAAV1-HGF significantly improved hindlimb strength and rotarod performance from 10 days after nerve crush, and these effects were maintained until the end of the experiment on day 28. These results suggested that rAAV1-HGF could promote functional recovery after sciatic nerve crush when delivered into the spinal cord.

It is well established that when the sciatic nerve is injured, Wallerian degeneration occurs and axons regenerate again as time goes by [42]. Therefore, we used IHC to examine whether rAAV1-HGF had effects on nerve regeneration in the areas of the nerve damage. Five days after nerve injury, the sciatic nerve was collected, and labeled with SCG10, a marker for the regenerating axon [43]. When the length of the regenerated sciatic nerve was measured, rAAV1-C showed a 2.11 ± 0.09 mm increase from the damaged site, while in the rAAV1-HGF group, it was 37.91% higher at 2.91 ± 0.27 mm **(**Fig. 2c-d**)**.

The presynaptic terminal of the sciatic nerve forms NMJs with the postsynaptic end plate of the muscle to transmit the contractile signal to the muscle [53]. Therefore, TA muscle connected to the sciatic nerve was analyzed by IHC. In a normal state, the NMJ is pretzel-shaped and has well-preserved integrity. After nerve injury, however, the shape of the NMJ becomes abnormal and the degree of integrity decreases (Fig. 2e-f). Compared with the sham group to which the nerve injury was not introduced, the proportion of fully innervated NMJs in the rAAV1-C group was reduced 2.34-fold, and that of abnormally shaped NMJs was increased 7.75-fold. On the other hand, in the rAAV1-HGF group, the proportion of fully innervated NMJs increased by 69.7%, and that of denervated NMJs decreased 3.83-fold in comparison to the rAAV1-C group **(**Fig. 2g-h**)**. The average α-BTX area was decreased by 41.75% in the rAAV1-C group compared to the sham group, but was increased by 56.76% in the rAAV1-HGF group. These results suggested that IT injection of rAAV1-HGF could promote the regeneration of neurons and re-establishment of NMJs possibly by acting on the cell body of motor neurons located in the spinal cord.

#### Effects of rAAV1-HGF in SOD1-G93A TG mouse model

Encouraged by the above results, the effects of rAAV1-HGF were also tested in the SOD1-G93A TG mouse model, the most commonly used animal model for ALS. First, we investigated whether HGF proteins expressed from rAAV1-HGF could also increase the phosphorylation of Met in SOD1-G93A TG mice. 5 × 10^9^ GC of rAAV1-HGF were intrathecally injected to SOD1-G93A TG mice at P60, and the levels of Met phosphorylation were measured 40 days later using IHC (Additional file 1: Figure S2a). In non-TG mice, 35.24 ± 0.55% of SMNs were co-stained with p-Met. The number of p-Met-positive cells was lowered in TG mice injected with a control vector, rAAV1-C, to 29.98 ± 0.54%, but increased to 59.65 ± 1.35% in the rAAV1-HGF group (Additional file 1: Figure S2b). These findings suggested that the HGF proteins expressed from rAAV1-HGF could indeed increase the phosphorylation of Met in this mouse model.

**Fig. 3:**
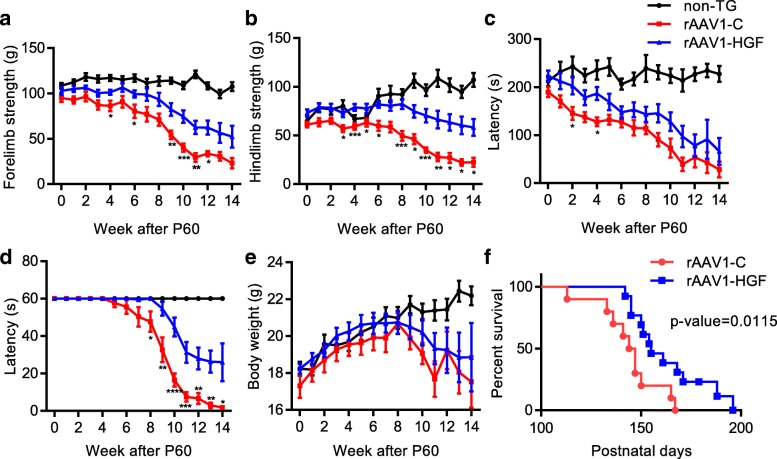
IT delivery of rAAV1-HGF ameliorated disease progression and prolonged the survival rate of SOD1-G93A TG mice. **a-f** SOD1-G93A TG mice at P60 were intrathecally injected with 5 × 10^11^ GC of rAAV1-C or rAAV1-HGF. Behavioral tests including those for forelimb strength (**a**), hindlimb strength (**b**), rotarod (**c**), and hanging wire (**d**) were performed, and body weight (**e**) was measured once a week until P158. In Fig. 3a-e, one-way ANOVA was performed followed by Tukey’s post-hoc test at each time point. ^*^p < 0.05, ^**^p < 0.01, ^***^p < 0.001, ^****^p < 0.0001. **f** Survival curve was drawn based on Mantel-Cox and Gehan-Breslow-Wilcoxon tests for comparing two groups. In the Gehan-Breslow-Wilcoxon test, *p* value was 0.0115. Median survival days were 145.5 for the rAAV1-C-treated group, and 155 for the rAAV1-HGF-treated group. For graphs, values are represented as mean ± SEM

To test whether rAAV1-HGF could exert any effect on disease progression of SOD1-G93A TG mice, body weight was measured and behavioral tests were performed once every week until P158 after injecting rAAV1-HGF into the LSC of TG mice at P60. IT delivery of rAAV1-HGF did not affect the rate of weight loss (Fig. 3e), but significant improvements were observed in rotarod, hanging wire, and grip strength tests **(**Fig. 3a-d**)**. In addition, survival rate was increased from 20 to 66.67% at P150, and median survival was also increased by 6.53% from 145.5 days to 155 days **(**Fig. 3f**)**. These results suggested that IT delivery of rAAV1-HGF might slow disease progression and improve survival rate in this mouse model.

### Effects of rAAV1-HGF on SMNs and NMJs in the SOD1-G93A TG mouse model

**Fig. 4:**
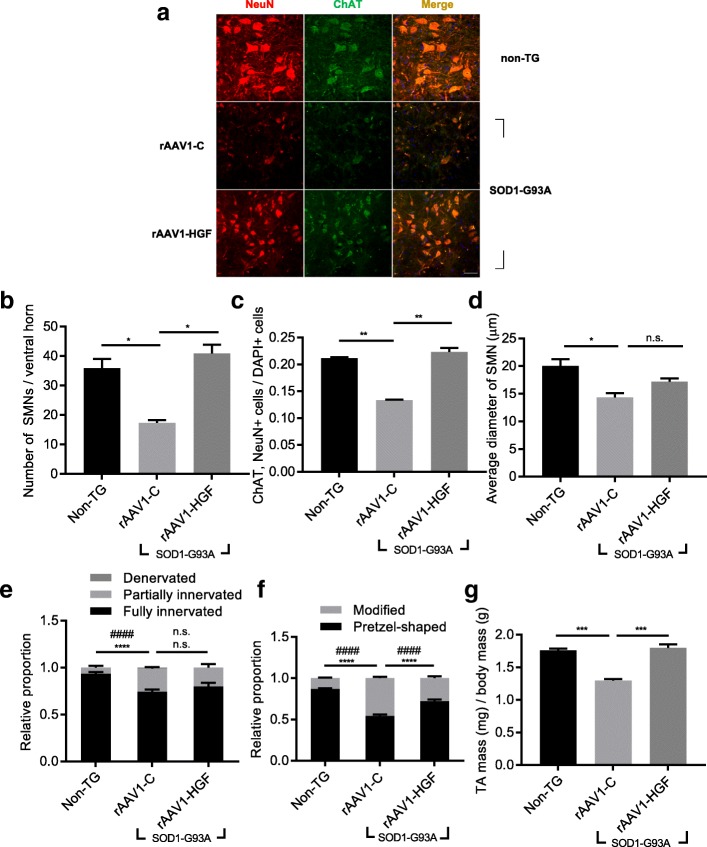
IT delivery of rAAV1-HGF led to histological improvements of SMNs and NMJs in SOD1-G93A TG mice. **a-g** non-TG or TG mice at P60 were intrathecally injected with rAAV1-C or rAAV1-HGF. The LSCs (**a-d**) and TA muscles (**e-f**) were collected at P100, followed by IHC assay. **a** Representative image of SMNs. Antibodies specific to ChAT and NeuN were used to label SMNs. **b** Number of SMNs per ventral horn was counted and represented as a bar graph. **c** Proportion of SMNs per DAPI-positive cells was counted and represented as a bar graph. **d** Diameter of SMNs were measured and represented as a bar graph. In, Fig. 4b-d, one-way ANOVA was performed, followed by Tukey’s post-hoc test. **e-f** The shape and integrity of NMJs were determined as mentioned in Fig. 2 and represented as a bar graph. In Fig. 4e-f, two-way ANOVA was performed, followed by Tukey’s post-hoc test. In Fig. 4e, ^****^p < 0.0001 for modified NMJ and ^####^*p* < 0.0001 for pretzel-shaped NMJ. In Fig. 4f, ^****^p < 0.0001 for fully innervated NMJ and ^####^*p* < 0.0001 for partially innervated NMJ. **g** Relative mass of TA was calculated using total body mass and represented as a bar graph. For bar graphs, values are represented as mean ± SEM. For statistical analysis, one-way ANOVA was performed, followed by Tukey’s post-hoc test. For bar graphs, ^*^p < 0.05, ^**^p < 0.005, ^***^p < 0.001, *n.s.* > 0.05. Scale bar: **a** = 50 μm

We tested whether IT injection of rAAV1-HGF could delay degeneration of SMNs in SOD1-G93A TG mice. 5 × 10^9^ GC of rAAV1-HGF were injected into the LSCs of SOD1-G93A TG mice at P60, and the LSCs were collected 40 days later (P100) followed by IHC. When compared to the non-TG group, the rAAV1-C group showed the number of SMNs in the ventral horn reduced 2.07-fold, the ratio of SMNs to total cells decreased by 58.65%, and the diameter of SMNs diminished by 39.83% **(**Fig. 4a**)**. Although rAAV1-HGF did not increase the diameter of SMNs, the number of SMNs was increased 2.36-fold and the ratio of SMNs to total cells was increased by 67.11%, compared to the rAAV1-C **(**Fig. 4b-d**)**.

We also examined whether changes in SMNs by the rAAV1-HGF could lead to improvements in connected axon terminals and muscles. Similar to the experiments described above (Fig. 2e-h), the NMJ was observed and the weight of TA was measured. Compared to the non-TG group, in the rAAV1-C group, the proportion of fully innervated NMJs was decreased by 25.48%, the level of abnormal-shaped NMJs was increased 3.5-fold, and the weight of TA was decreased by 35.42%. When rAAV1-HGF was injected, however, the proportion of pretzel-shaped NMJs was increased by 32.58% (Fig. 4e-f), and the weight of TA, which was decreased by muscular atrophy in the rAAV1-C group, was increased by 38.42% (Fig. 4g). No visible change was observed in the degree of NMJ integrity in the rAAV1-HGF group. These results indicated that IT delivery of rAAV1-HGF could promote the protection of peripheral nerves and NMJs as well as SMNs.

### Effects of rHGF on axonal outgrowth of CSMNs

The above data showed that IT delivery of rAAV1-HGF could improve motor functions and survival rates of SOD1-G93A TG mice, presumably by slowing down the degeneration of SMNs and restoring the morphology of NMJs. Since the interaction of HGF with lower motor neurons has been relatively well established compared to the interaction of HGF with upper motor neurons [8, 54, 56], we were interested in testing the possible involvement of upper motor neurons by using the motor cortical cultures system consisting of CSMNs and glial cells [18, 35].

**Fig. 5:**
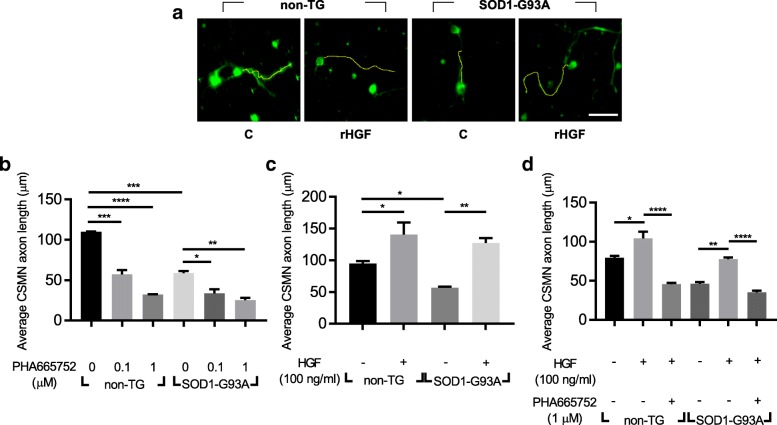
Treatment with rHGF enhanced the axonal outgrowth of CSMNs in motor cortical cultures. **a-d** SOD1-G93A TG mice were sacrificed at P3. After dissociating motor cortices, 20,000 cells were seeded on 24-well plates. Three days later, cells were fixed and subjected to immunocytochemistry (ICC) assay. **a** Representative image of CSMNs. Antibodies specific to UCHL1 (green) and Ctip2 (red, not shown) were used to label CSMNs. Serum-free media was used as a control medium (C). Cells were visualized with confocal laser scanning microscopy. The axon length of CSMNs was measured using Fiji software (yellow line). The axon length of CSMNs was measured after treatment with PHA665752, an inhibitor for Met (**b**) or rHGF (**c**) or rHGF plus PHA665752 (**d**). For bar graphs, values are represented as mean ± SEM. In Fig. 5b-d, one-way ANOVA was performed, followed by Tukey’s post-hoc test. ^*^p < 0.05, ^**^p < 0.01, ^***^p < 0.001, ^****^p < 0.0001. Scale bar: **a** = 50 μm

The motor cortices were isolated from P3 non-TG or TG mice, and 2 × 10^4^ cells were cultured on a 24-well plate. Three days later, cells were fixed, followed by IHC analysis. CSMNs were labeled with UCHL1 and Ctip2, and Fiji software was used to measure the axon length of CSMNs [58] **(**Fig. 5a**)**.

Since CSMNs have previously been shown to produce HGF which may give experimental noise, the effects of endogenously expressed HGF were first tested using PHA665752, a chemical inhibitor of Met. When cells were treated with 1 μM of PHA665752 for 3 days, the axon length of CSMNs was reduced 3.42-fold in the non-TG group and 2.33-fold in the TG group **(**Fig. 5b**)**. This result indicated that endogenously expressed HGF could positively affect the axonal outgrowth of CSMNs.

When 100 ng/ml of rHGF were added to the culture for 3 days, however, the axon length of CSMNs was increased by 48.61% in the non-TG group, and by 124.73% in the TG group **(**Fig. 5c**)**. When PHA665752 was co-treated with rHGF, the HGF-mediated increase of CSMNs’ axon length was inhibited by 127.7% in the non-TG group, and by 120.82% in the TG group **(**Fig. 5d**)**. This is consistent with the data in Fig. 7a-b showing activation or inhibition of phosphorylation of the Met protein by HGF or PHA665752. These results suggested that activation of the HGF-Met signaling pathway might be involved in the promotion of axonal outgrowth of CSMNs.

### Effects of inhibition of ERK, PI3K, and p38 on axonal outgrowth of CSMNs

**Fig. 6:**
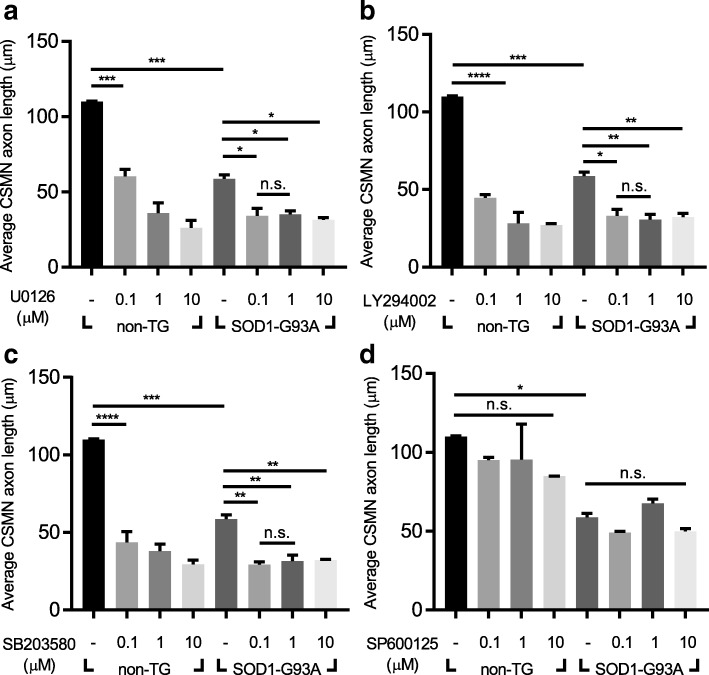
Inhibition of ERK, PI3K, and p38 resulted in decreased axonal outgrowth of CSMNs. **a-d** The axon length of CSMNs was measured and represented as a bar graph after treatment with U0126 (ERK inhibitor) (**a**), LY294002 (PI3K inhibitor) (**b**), SB203580 (p38 inhibitor) (**c**), and SP600125 (JNK inhibitor) (**d**). For bar graphs, values are represented as mean ± SEM. In Fig. 6a-d, one-way ANOVA was performed, followed by Tukey’s post-hoc test. ^*^p < 0.05, ^**^p < 0.01, ^***^p < 0.001, ^****^p < 0.0001, *n.s.* > 0.05

Several signaling pathways have been shown to be turned on upon interaction between HGF and Met receptor. To identify the key pathway involved in the regulation of the HGF-mediated axonal outgrowth of CSMNs, 2 × 10^4^ cells were treated with specific chemical inhibitors for ERK (U0126), PI3K (LY294002), p38 (SB203580), and JNK (SP600125). When cells were treated with 3 different concentrations of respective inhibitors, the axon length of CSMNs was reduced in a dose-dependent manner by all agents except for SP600125 **(**Fig. 6a-d**)**. In all concentrations of chemical inhibitors used in this experiment, cytotoxic effects were not observed (Additional file 1: Figure S3a). These results suggested that ERK, PI3K, and p38 might play roles in the HGF-mediated axonal outgrowth of CSMNs.

### Effects of rHGF on ERK phosphorylation

**Fig. 7:**
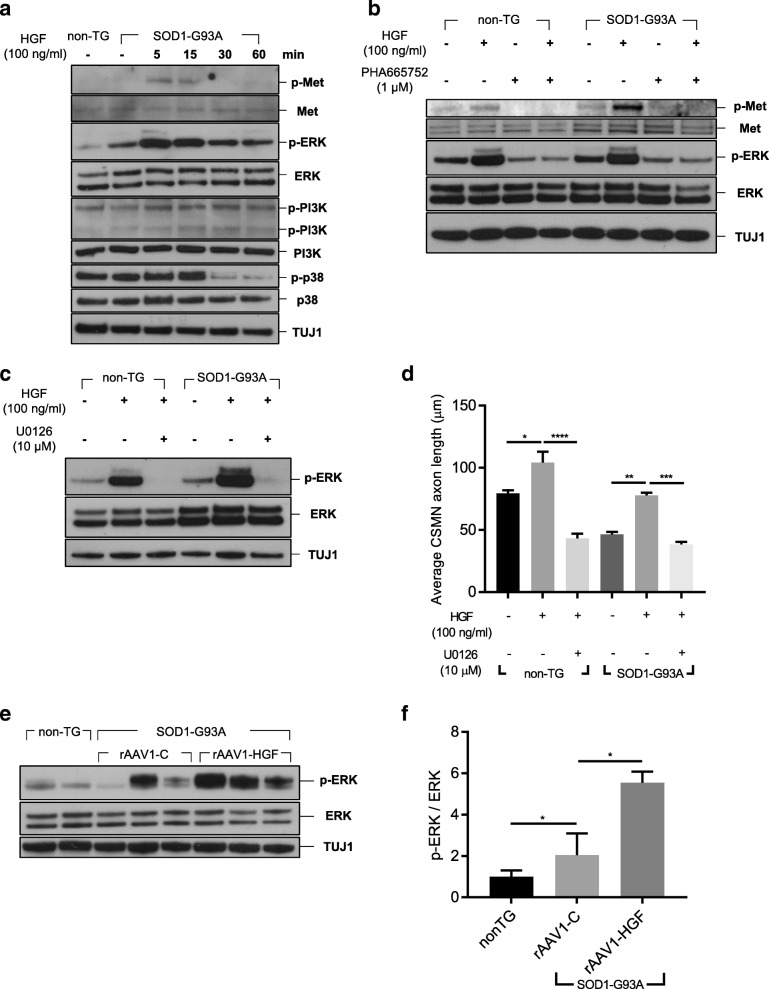
Phosphorylation of ERK was increased by treatment with HGF in the LSC and motor cortical cultures. **a-c** Motor cortices from non-TG or TG mice at P3 were collected. After dissociation, cells were seeded on six-well plates with 3.2 × 10^6^ cells/well. **a** Cells were treated with 100 ng/ml of rHGF, and 5 days later, total proteins were isolated, followed by Western blot analysis. **b-c** Cells were treated with 1 μM of PHA665752 or 10 μM of U0126. After 30 min, cells were treated with 100 ng/ml of rHGF, and five days later, total proteins were isolated followed by Western blot analysis. DMSO was used as a negative control. **d** The axon length of CSMNs was measured and represented as a bar graph after co-treatment with rHGF and U0126. For statistical analysis, one-way ANOVA was performed, followed by Tukey’s post-hoc test. **e-f** non-TG or TG mice at P60 were intrathecally injected with rAAV1-C or rAAV1-HGF. The LSCs were collected, and total proteins were extracted at P100, followed by Western blot analysis (**e**). Relative levels of phosphorylated ERK were measured using Fiji software and represented as a bar graph (**f**). For bar graphs, values are represented as mean ± SEM. For statistical analysis, one-way ANOVA was performed, followed by Tukey’s post-hoc test. ^*^p < 0.05, ^**^p < 0.01, ^***^p < 0.001, ^****^p < 0.0001

We tested whether HGF could activate ERK, PI3K, and p38 in the cortical culture. 3.2 × 10^6^ cells were treated with 100 ng/ml of rHGF, and 5 days later, total proteins were extracted, followed by Western blot analysis. As shown in Fig. 7a, the level of phosphorylated ERK was increased by treatment with rHGF, while the levels of PI3K and p38 were not affected. The effect of rHGF was inhibited when cells were co-treated with 1 μM of PHA665752 or 10 μM of U0126 **(**Fig. 7b-c**)**, suggesting that the HGF-Met pathway is involved in the phosphorylation of ERK in this cortical culture. We tested whether inhibition of ERK could reduce the HGF-mediated increase of the axon length of CSMNs. As shown in Fig. 7d, treatment with U0126 decreased the axon length of CSMNs 2.41-fold in the non-TG group and 2.03-fold in the TG group, indicating that HGF could promote axonal outgrowth of CSMNs by specifically up-regulating phosphorylation of ERK.

To test whether the above in vitro results were reproducible in vivo, 5 × 10^9^ GC of viral vectors were intrathecally injected into SOD1-G93A TG mice at P60, and the LSC was analyzed for ERK. Compared with non-TG mice, the level of phosphorylated ERK was increased 2.04-fold in TG mice injected with a control vector, and was further enhanced 5.54-fold when injected with AAV vectors expressing HGF **(**Fig. 7e-f**)**. There was no difference in phosphorylation of other signaling molecules of the HGF-Met pathway, such as STAT3, cJUN, and GSK3β (Additional file 1: Figure S4a). These results indicated that ERK might indeed be an important factor of the HGF-mediated regeneration of motor neurons.

### Effects of HGF-mediated ERK phosphorylation on levels of ROS

**Fig. 8:**
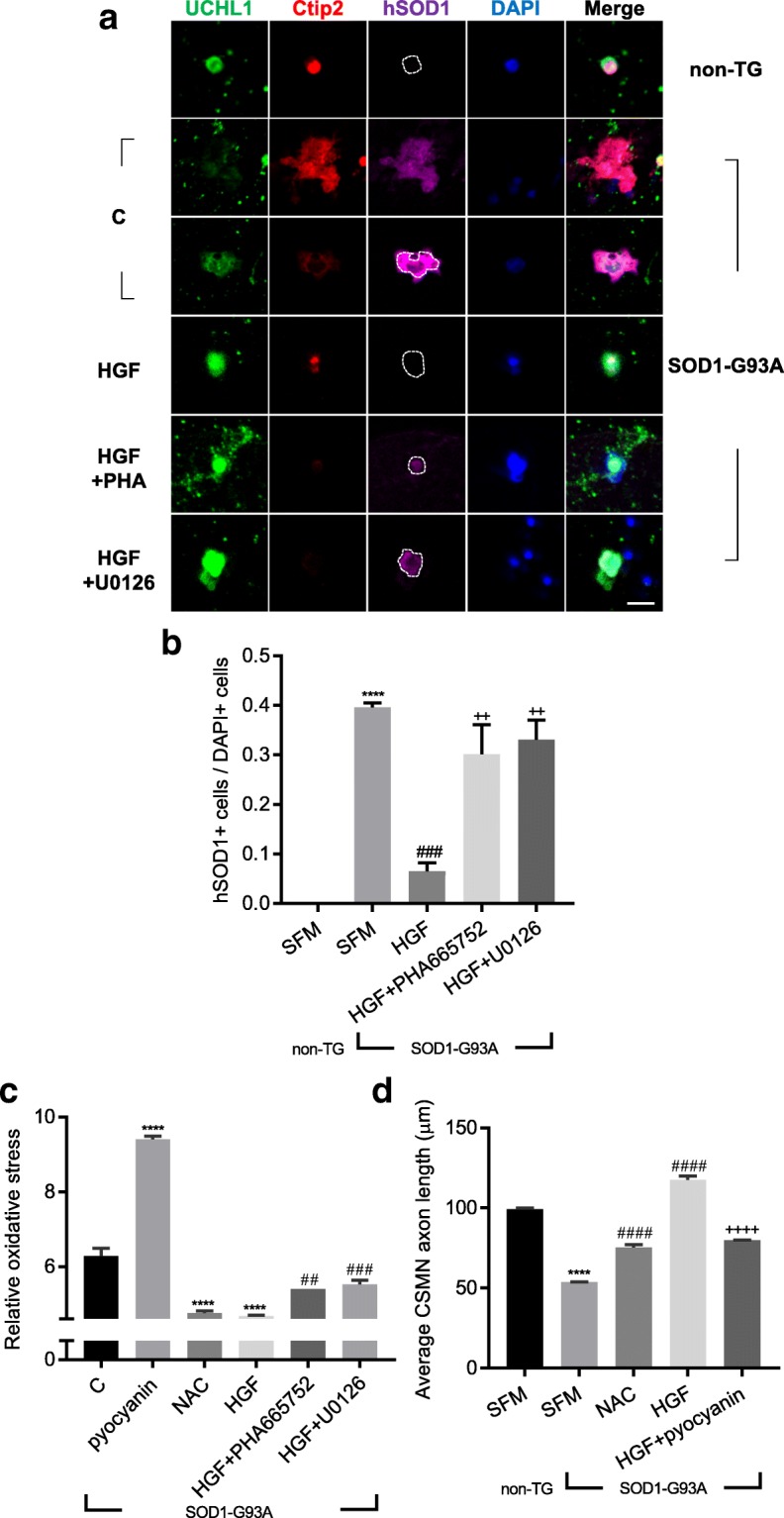
Treatment with rHGF reduced levels of protein aggregation and oxidative stress induced by mutant SOD1. **a-b** ICC assay was performed as described in Fig. 5a. Antibodies specific to UCHL1 and Ctip2 were used to label CSMNs, together with those for hSOD1 (magenta). C: Treated with SFM. In the hSOD1-stained panels, the cell boundaries of the CSMNs were outlined based on the UCHL1 signals, except for the panel in the second row, where the cell boundaries are ambiguous. Proportion of hSOD1-positive cells per DAPI-positive cells was counted and represented as a bar graph (**b**). For statistical analysis, one-way ANOVA was performed, followed by Tukey’s post hoc test. ^****^p < 0.0001 for non-TG SFM vs. TG SFM, ^###^p < 0.001 for TG SFM vs. TG HGF, ^++^*p* < 0.01 for TG HGF vs. TG HGF + PHA665752 (or TG HGF + U0126). **c** 6.4 × 10^4^ cells were seeded on 96-well plates. Cellular ROS levels were measured using a ROS detection kit and represented as a bar graph. Pyocyanin was used as an inducer of ROS, whereas N-acetyl-L-cysteine (NAC) was employed as a scavenger of ROS. For statistical analysis, one-way ANOVA was performed, followed by Tukey’s post-hoc test. ^****^p < 0.0001 for SFM vs. pyocyanin (or NAC or HGF), ^##^*p* < 0.01 for HGF vs. HGF + PHA665752, ^###^*p* < 0.001 for HGF vs. HGF + U0126. **d** The axon length of CSMNs was measured and represented as a bar graph. For bar graphs, values are represented as mean ± SEM. For statistical analysis, one-way ANOVA was performed, followed by Tukey’s post-hoc test. ^****^p < 0.0001 for non-TG SFM vs. TG SFM, ^####^p < 0.0001 for TG SFM vs. TG NAC (or TG HGF), ^++++^*p* < 0.0001 for TG HGF vs. TG HGF + pyocyanin. Scale bar: **a** = 20 μm

To get an overall picture of the effects of HGF on gene expression profile in CSMNs, cortical cells were treated with 100 ng/ml of rHGF, and total RNAs were extracted followed by microarray assay (Additional file 1: Figure S5a). Among the 116 genes whose expression levels were changed more than 1.5-fold, 52 belonged to six major categories defined for fALS by Taylor et al., and 37 (71.15%) were involved in the control of protein quality and RNA metabolism [49]. Therefore, we tested whether the HGF-Met-ERK signaling pathway could reduce hSOD1 protein aggregation and/or oxidative stress. Cortical cells were treated with rHGF, followed by IHC to examine the distribution of mutant hSOD1. When compared to the non-TG group containing no hSOD1 aggregates, the proportion of CSMNs in the TG group with hSOD1 aggregates was sharply increased to 0.4 ± 0.01 as shown in Fig. 8a-b. When treated with rHGF, however, this proportion was reduced to 0.06 ± 0.02, while it was increased to 0.3 ± 0.06 and 0.33 ± 0.04 by the addition of inhibitors for Met (PHA665752) or ERK (U0126), respectively **(**Fig. 8a**)**.

Since the aggregated form of mutant hSOD1 could increase oxidative stress, it was also tested whether the rHGF-mediated reduction of protein aggregation could alleviate oxidative stress by measuring hydrogen peroxide, peroxynitrite, hydroxyl radicals, nitric oxide, and peroxy radical in the cortical culture. Compared to the control group treated with SFM, treatment with rHGF decreased the level of oxidative stress by 34.22%, which was comparable to the effect (32.11%) of N-acetyl-L-cysteine (NAC), a well-known scavenger of oxygen free radical **(**Fig. 8c**)**. Such HGF-mediated decrease of oxidative stress was inhibited by 15.51 and 18.81% when rHGF was co-treated with PHA665752 and U0126, respectively **(**Fig. 8c**)**.

To test whether the axonal outgrowth of CSMNs could be promoted by alleviation of oxidative stress, the effect of rHGF was tested in the presence of pyocyanin, a ROS inducer. As shown in Fig. 8d, the axon length of CSMNs was increased by 40.12% when treated with NAC. The effect of rHGF was almost 3-fold greater at 118.83%, which was inhibited by 32.1% in the presence of pyocyanin. Taken together, these results indicated that HGF-mediated induction of ERK phosphorylation plays an important role(s) in promoting the axonal outgrowth of CSMNs by mitigating protein aggregation and oxidative stress.

## Discussion

In this report, we explored the possibility of delivering the HGF gene by IT injection of rAAV vector for neuromuscular diseases. It was found that AAV serotype 1 could most effectively deliver and express the transgene in the ventral horn. rAAV1-HGF produced the HGF protein in a bell shape kinetics pattern over the course of 16 weeks, with peak level achieved at 2 weeks post-injection. In two neuromuscular disease models—the sciatic nerve crush and SOD1-G93A TG mouse models—a single administration of rAAV1-HGF into the LSC improved motor functions and NMJ structure. These results indicated that rAAV1 expressing HGF might have regenerative potential and improve symptomatic motor performance.

Of particular interest was the finding that IT injection of rAAV1-HGF could not only facilitate protection of but also delay the degeneration of motor neurons. In the sciatic nerve crush model, for example, the regenerative process takes place after Wallerian degeneration following nerve injury, and rAAV1-HGF was shown to promote the regeneration of the sciatic nerves and the recovery of NMJ structure. On the other hand, in the SOD1-G93A TG mouse model, progressive degeneration of motor neurons and muscles occurs, and rAAV1-HGF could delay disease progression and degeneration of SMNs. Therefore, rAAV1-HGF appears to provide dual activities in the pathogenesis of motor neuron degeneration that may result in additive effects.

Our results are consistent with previous studies showing the potentially positive roles of HGF or Met in ALS patients or related animal models. Protein levels of HGF and Met increase in the ventral horn of sALS patients at the early stage, while patients with motor neurons defective in HGF or Met are more susceptible to disease progression of ALS, being atrophied rapidly [20]. In SOD1-G93A TG mice, RNA levels of HGF and Met were higher than the non-TG control, presumably to respond to or compensate for pathological conditions [48]. In the same model, disease progression was shown to be delayed and the survival rate to be increased when either HGF or Met were overexpressed specifically in motor neurons or when rHGF was injected into the spinal cord. Together with our results, HGF appears to be a strong candidate that may be used for developing therapeutics for ALS.

Although protein levels of HGF and Met have been shown to increase in SOD1-G93A TG mice, the level of biologically active forms of these proteins has not been well studied in the LSC at the symptomatic stage. We found that the level of phosphorylated Met was slightly lower in SMNs of the ventral horn of SOD1-G93A TG mice at P100, indicating that the level of endogenously expressed HGF might not be sufficient to fully activate Met. When TG mice were intrathecally injected with rAAV1-HGF, however, the fraction of SMNs expressing phosphorylated Met approximately doubled, suggesting that exogenously added HGF could augment the activation of Met. Therefore, HGF appeared to produce cell-autonomous effects by directly affecting SMNs. HGF is also expected to generate non-autonomous effects by acting on glial cells such as astrocytes and microglia. We previously showed that HGF produced from plasmid DNA expression vector could lower the level of ATF3 and CSF1 in DRG and change the distribution of activated microglia in the dorsal horn in the mouse chronic constriction injury model [33]. Others also reported that treatment of primary astrocytes with HGF in vitro reduced the protein level of EAAT2, a glutamate transporter, and enhance the expression level of total Met in reactive astrocytes in the spinal cord [20, 48]. We also observed that treatment with rHGF could lower the levels of LPS-induced TNFα in the primary astrocyte culture system (data not shown). Together with the cell-autonomous effects described above, HGF has, when properly delivered to patients, the potential to generate powerful therapeutic effects by controlling two of the most important pathways in the pathogenesis of ALS and related motor neuron diseases.

Improvements in motor functions, NMJ morphology, and the protection of SMNs observed in this study could have resulted from 3 sources: changes in SMNs, in upper motor neurons, or in a combination of both neurons. HGF is known as a potent neurotrophic factor, which can facilitate the proliferation, migration, differentiation, and survival of sensory neurons, as well as motor neurons [19, 26, 27, 34]. The interaction of HGF with lower motor neurons has been relatively well characterized. For example, it has been reported that HGF could function as a survival factor for SMNs in vitro [8, 54, 56]. In addition, HGF has also been shown to induce remyelination of Schwann cells to promote axonal outgrowth of peripheral neurons [24]. These findings suggested that HGF could directly and/or indirectly protect damaged lower motor neurons.

On the other hand, the molecular mechanisms of HGF’s interaction with upper motor neurons have been poorly understood up until now. In this study, therefore, we investigated the effects of HGF on upper motor neurons in vitro, using the primary motor cortical culture system consisting of CSMNs and glial cells. It was observed that HGF could facilitate the axonal outgrowth of CSMNs by controlling the phosphorylation of ERK. When phenotypes of oxidative stress were analyzed based on microarray data, treatment of CSMNs with HGF protein was found to reduce the accumulation of mutant SOD1 proteins and levels of oxidative stress factors such as H_2_O_2_, ONOO^−^, HO, NO, and ROO. All these effects were inhibited, however, when ERK phosphorylation was suppressed by U0126. These results suggested that upper motor neurons could also have played a role in the effects exerted by rAAV1-HGF, and that ERK might be a key signaling factor involved.

It is also possible that HGF expressed from rAAV1-HGF could have affected both spinal and upper motor neurons, so observed improvements in the SOD1-G93A TG mouse model might have come from the combined effects. Our preliminary data indicated that intramuscular injection of rAAV6-HGF into the tibialis anterior did not have particularly visible effects on motor functions and other parameters (data not shown). Therefore, we are inclined to think that the role of upper motor neurons may have been more important in this particular model.

In summary, our data suggested that exogenously added HGF, in the form of rAAV1-HGF intrathecally delivered to the LSC area, could improve behavioral defects and survival rates in SOD1-G93A TG mice. ERK activated by HGF from rAAV1-HGF appeared to play a key role(s) in HGF-mediated effects such as alleviation of oxidative stress and facilitation of protection, and delay of degeneration of motor neurons. Taken together, it appears that rAAV1-HGF may be developed as a novel therapeutic agent for various diseases in which motor neuron degeneration is the major pathologic cause.

## Additional file

Additional file 1: **Figure S1.** Production of HGF-expressing rAAV vector. **a** To co-express two isoforms of HGF, HGF723 (or dHGF) and HGF728 (or cHGF), a gDNA-cDNA-hybrid sequence was generated. In this chimeric sequence, a part of intron 4 of the HGF gene was inserted between cDNA sequences of exon 4 and exon 5, allowing alternative splicing. Since the length of intron 4 is relatively long, sequences between 246 and 4486 were deleted (Δ). The numbers represented indicate relative positions of intron 4, and ‘1’ corresponds to the first nucleotide of intron 4. **b** 1.6x10^5^ C2C12 cells were transduced with 1.23x10^8^ GC of rAAV2, and 48 hours later, total RNAs were isolated followed by RT-PCR and acrylamide gel analysis. rAAV2-C lacking the HGF sequence was used as a negative control (NC). The upper arrow indicates the amplicon size of cHGF (142 bp), while the lower arrow shows the amplicon size of dHGF (127 bp). **c** 8x10^4^ C2C12 cells were transduced with 5x10^13^ GC of rAAV2, and 48 hours later, supernatant was collected followed by ELISA for hHGF. rAAV2-C lacking the HGF sequence was used as a negative control. ND indicates that values were not detectable or lower than the minimum detectable dose. **d** C57BL/6 mice at P60 were intrathecally injected with 5x10^9^ GC of rAAV1-C or rAAV1-HGF. The LSCs, motor cortices, serum, and TA were collected 8 weeks after injection and subjected to ELISA for hHGF. For bar graphs, values are represented as mean ± SEM. **Figure S2.** Levels of phosphorylated Met after IT delivery of rAAV1-HGF. **a** non-TG or TG mice at P60 were intrathecally injected with 5x10^9^ GC of rAAV1-C or rAAV1-HGF. The LSCs were collected at P100. Tissues were fixed and subjected to IHC assay. Antibodies specific to ChAT (green) and NeuN (red) were used to label SMNs, together with those for p-Met (magenta). **b** The proportion of SMNs expressing p-MET per total SMNs was measured and represented as a bar graph. For bar graphs, values are represented as mean ± SEM. Scale bar: **a** = 20 µm. *p < 0.05, n.s. > 0.05. **Figure S3.** Effects of rHGF or chemical inhibitors measured by WST1 assay in motor cortical cells. **a** After dissociating motor cortices of P3 non-TG or TG mice, 1.6x10^5^ cells were seeded on PDL-coated 48-well cell plates in the presence of rHGF or respective inhibitors. Three days later, WST1 assay was performed. Cell viability was measured using a microplate reader and represented as a bar graph. For the bar graph, values are represented as mean ± SEM. n.s. > 0.05.  **Figure S4.** Western blot analysis in the LSC after IT delivery of rAAV1-HGF. **a** non-TG or TG mice at P60 were intrathecally injected with 5 × 10^9^ GC of rAAV1-C or rAAV1-HGF. The LSCs were collected at P100. Total proteins were extracted and subjected to Western blot analysis. **Figure S5.** Microarray analysis of motor cortical cells treated with rHGF. **a** Motor cortical cells were treated with 100 ng/ml of rHGF for 3 days, followed by microarray analysis using Affymetrix Genechip. SFM was used as a negative control (NC). After data extraction, RMA normalization was performed followed by DEG analysis. Differential expression was represented by color gradients. Genes were clustered based on 6 major categories defined by Taylor et al., as described in Results. Genes involved in protein quality control are labeled in red, while those related to RNA metabolism are labeled in blue. The rest of genes are labeled in black. The NC group is for non-TG NC vs. TG NC, whereas the rHGF group is for TG NC vs. TG rHGF. **Table S1.** Antibodies and primers used in this study. **a-b** List of antibodies used for Western blot analysis (**a**) and immunostaining (**b**). **c** List of primers used for murine IL2 and human SOD1 to determine the copy number of mhSOD1. F: Forward primer, R: Reverse primer. (PPTX 7050 kb)
