# Supplementary material for: Intrathecal delivery of recombinant AAV1 encoding hepatocyte growth factor improves motor functions and protects neuromuscular system in the nerve crush and SOD1-G93A transgenic mouse models
Source: Acta Neuropathol Commun. 2019 Jun 12;7:14. doi: 10.1186/s40478-019-0737-z (PMC6563368; doi:10.1186/s40478-019-0737-z)
Supplement: Supplementary file 1 — Figure S1. Production of HGF-expressing rAAV vector. a To co-express two isoforms of HGF, HGF723 (or dHGF) and HGF728 (or cHGF), a gDNA-cDNA-hybrid sequence was generated. In this chimeric sequence, a part of intron 4 of the HGF gene was inserted between cDNA sequences of exon 4 and exon 5, allowing alternative splicing. Since the length of intron 4 is relatively long, sequences between 246 and 4486 were deleted (Δ). The numbers represented indicate relative positions of intron 4, and ‘1’ corresponds to the first nucleotide of intron 4. b 1.6x10^5^ C2C12 cells were transduced with 1.23x10^8^ GC of rAAV2, and 48 hours later, total RNAs were isolated followed by RT-PCR and acrylamide gel analysis. rAAV2-C lacking the HGF sequence was used as a negative control (NC). The upper arrow indicates the amplicon size of cHGF (142 bp), while the lower arrow shows the amplicon size of dHGF (127 bp). c 8x10^4^ C2C12 cells were transduced with 5x10^13^ GC of rAAV2, and 48 hours later, supernatant was collected followed by ELISA for hHGF. rAAV2-C lacking the HGF sequence was used as a negative control. ND indicates that values were not detectable or lower than the minimum detectable dose. d C57BL/6 mice at P60 were intrathecally injected with 5x10^9^ GC of rAAV1-C or rAAV1-HGF. The LSCs, motor cortices, serum, and TA were collected 8 weeks after injection and subjected to ELISA for hHGF. For bar graphs, values are represented as mean ± SEM. Figure S2. Levels of phosphorylated Met after IT delivery of rAAV1-HGF. a non-TG or TG mice at P60 were intrathecally injected with 5x10^9^ GC of rAAV1-C or rAAV1-HGF. The LSCs were collected at P100. Tissues were fixed and subjected to IHC assay. Antibodies specific to ChAT (green) and NeuN (red) were used to label SMNs, together with those for p-Met (magenta). b The proportion of SMNs expressing p-MET per total SMNs was measured and represented as a bar graph. For bar graphs, values are represented as mean ± SEM. Scale bar: a = 20 µm. *p < 0.05, n.s. > 0 [file 40478_2019_737_MOESM1_ESM.pptx]

## Slide 1
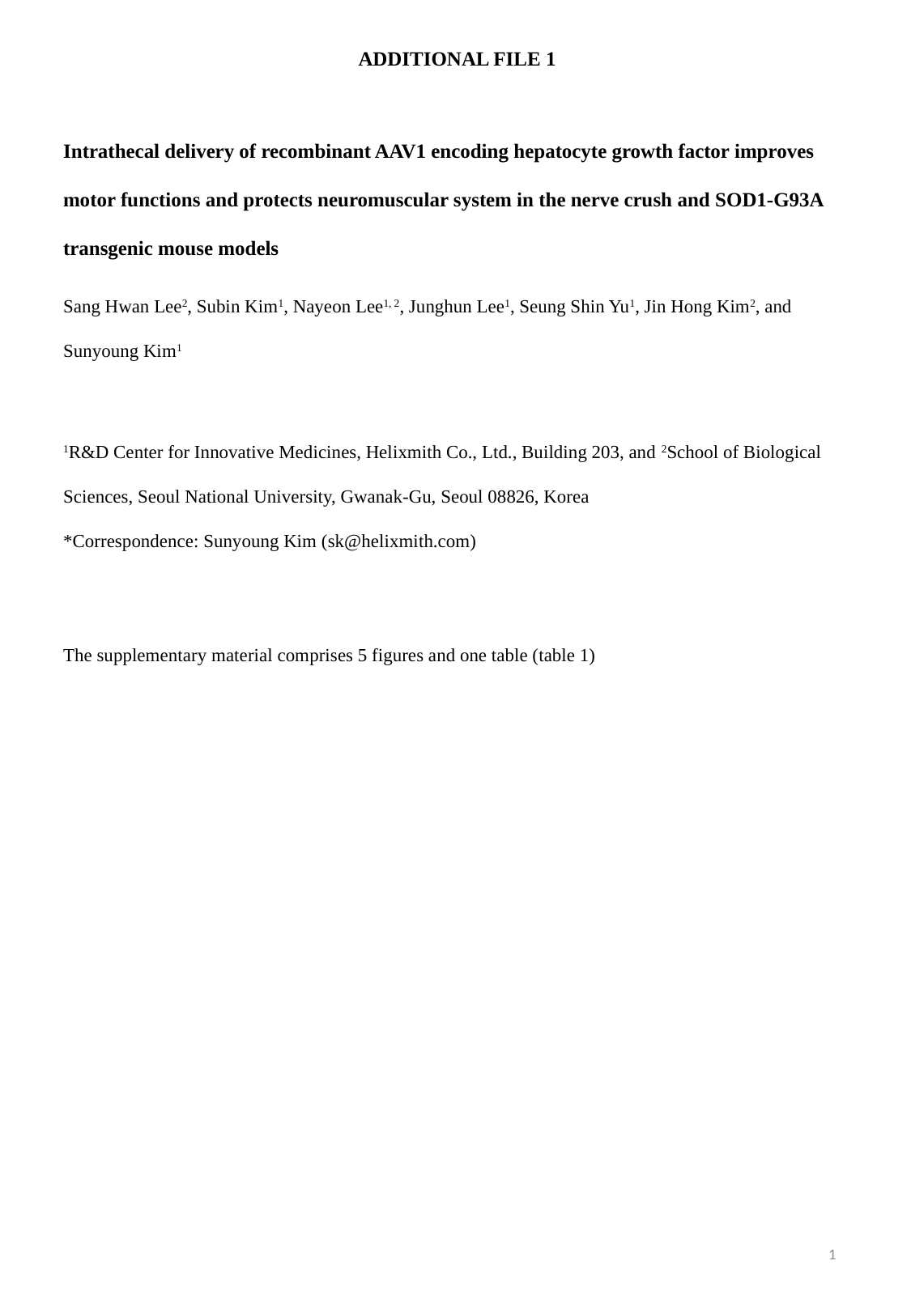

# ADDITIONAL FILE 1
Intrathecal delivery of recombinant AAV1 encoding hepatocyte growth factor improves motor functions and protects neuromuscular system in the nerve crush and SOD1-G93A transgenic mouse models
Sang Hwan Lee2, Subin Kim1, Nayeon Lee1, 2, Junghun Lee1, Seung Shin Yu1, Jin Hong Kim2, and Sunyoung Kim1
1R&D Center for Innovative Medicines, Helixmith Co., Ltd., Building 203, and 2School of Biological Sciences, Seoul National University, Gwanak-Gu, Seoul 08826, Korea
*Correspondence: Sunyoung Kim (sk@helixmith.com)
The supplementary material comprises 5 figures and one table (table 1)
1

## Slide 2
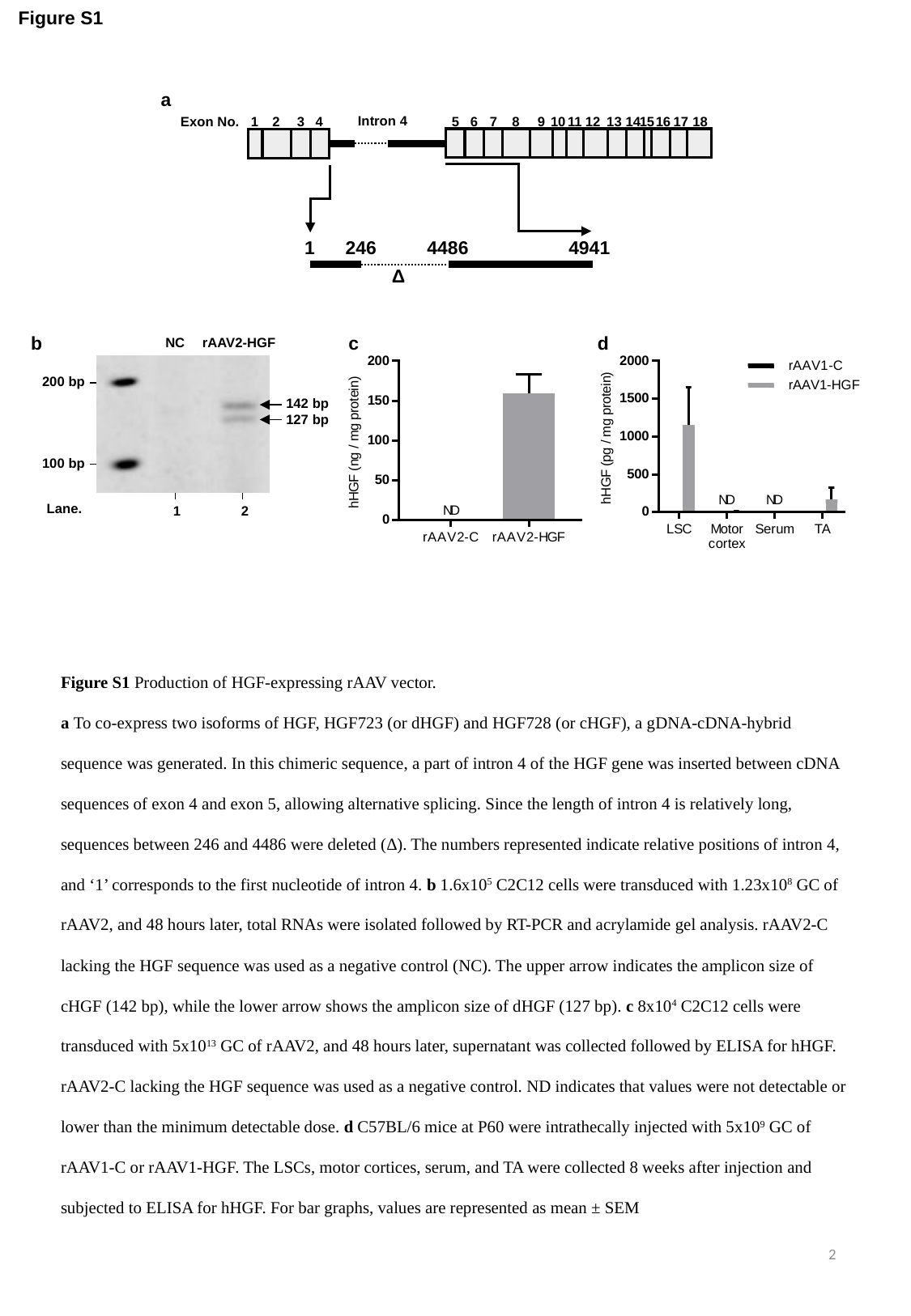

Figure S1
a
Intron 4
Exon No.
1
2
3
4
5
6
7
8
9
10
11
12
13
14
15
16
17
18
1
246
4486
4941
Δ
b
c
d
NC
rAAV2-HGF
200 bp
142 bp
127 bp
100 bp
Lane.
1
2
Figure S1 Production of HGF-expressing rAAV vector.
a To co-express two isoforms of HGF, HGF723 (or dHGF) and HGF728 (or cHGF), a gDNA-cDNA-hybrid sequence was generated. In this chimeric sequence, a part of intron 4 of the HGF gene was inserted between cDNA sequences of exon 4 and exon 5, allowing alternative splicing. Since the length of intron 4 is relatively long, sequences between 246 and 4486 were deleted (Δ). The numbers represented indicate relative positions of intron 4, and ‘1’ corresponds to the first nucleotide of intron 4. b 1.6x10^5^ C2C12 cells were transduced with 1.23x10^8^ GC of rAAV2, and 48 hours later, total RNAs were isolated followed by RT-PCR and acrylamide gel analysis. rAAV2-C lacking the HGF sequence was used as a negative control (NC). The upper arrow indicates the amplicon size of cHGF (142 bp), while the lower arrow shows the amplicon size of dHGF (127 bp). c 8x10^4^ C2C12 cells were transduced with 5x10^13^ GC of rAAV2, and 48 hours later, supernatant was collected followed by ELISA for hHGF. rAAV2-C lacking the HGF sequence was used as a negative control. ND indicates that values were not detectable or lower than the minimum detectable dose. d C57BL/6 mice at P60 were intrathecally injected with 5x10^9^ GC of rAAV1-C or rAAV1-HGF. The LSCs, motor cortices, serum, and TA were collected 8 weeks after injection and subjected to ELISA for hHGF. For bar graphs, values are represented as mean ± SEM
2

## Slide 3
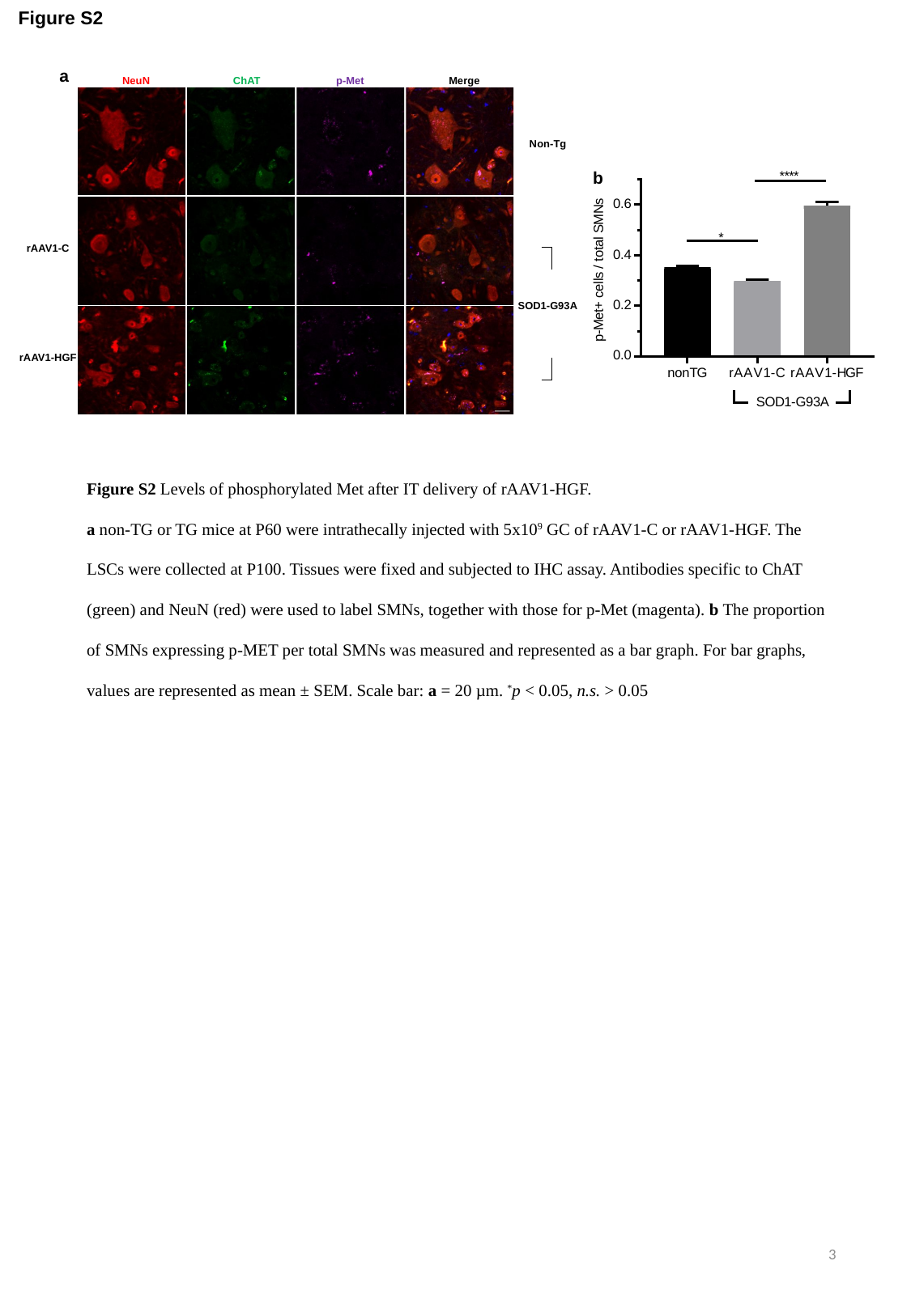

Figure S2
a
NeuN
ChAT
p-Met
Merge
Non-Tg
rAAV1-C
SOD1-G93A
rAAV1-HGF
b
Figure S2 Levels of phosphorylated Met after IT delivery of rAAV1-HGF.
a non-TG or TG mice at P60 were intrathecally injected with 5x10^9^ GC of rAAV1-C or rAAV1-HGF. The LSCs were collected at P100. Tissues were fixed and subjected to IHC assay. Antibodies specific to ChAT (green) and NeuN (red) were used to label SMNs, together with those for p-Met (magenta). b The proportion of SMNs expressing p-MET per total SMNs was measured and represented as a bar graph. For bar graphs, values are represented as mean ± SEM. Scale bar: a = 20 µm. *p < 0.05, n.s. > 0.05
3

## Slide 4
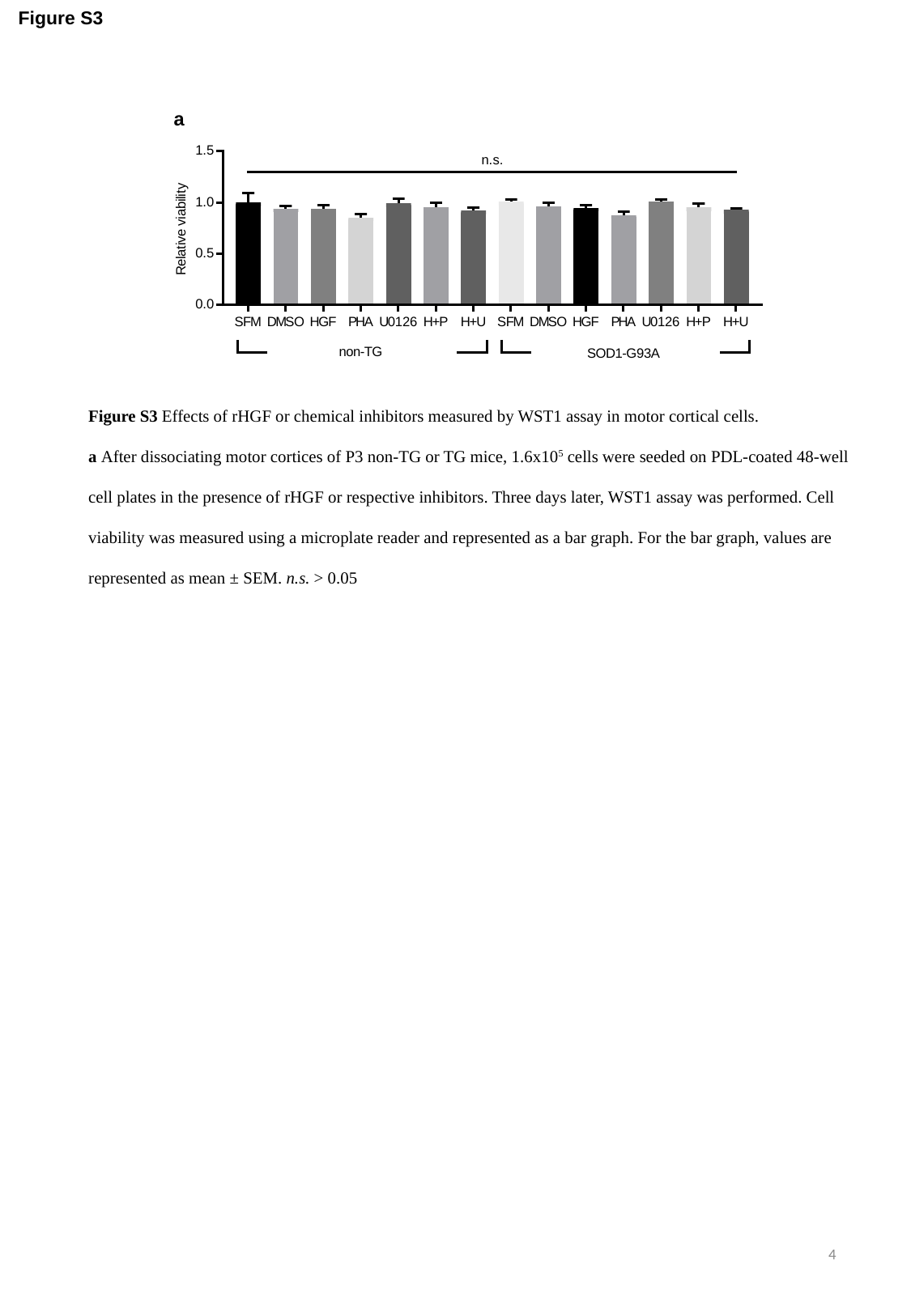

Figure S3
a
Figure S3 Effects of rHGF or chemical inhibitors measured by WST1 assay in motor cortical cells.
a After dissociating motor cortices of P3 non-TG or TG mice, 1.6x10^5^ cells were seeded on PDL-coated 48-well cell plates in the presence of rHGF or respective inhibitors. Three days later, WST1 assay was performed. Cell viability was measured using a microplate reader and represented as a bar graph. For the bar graph, values are represented as mean ± SEM. n.s. > 0.05
4

## Slide 5
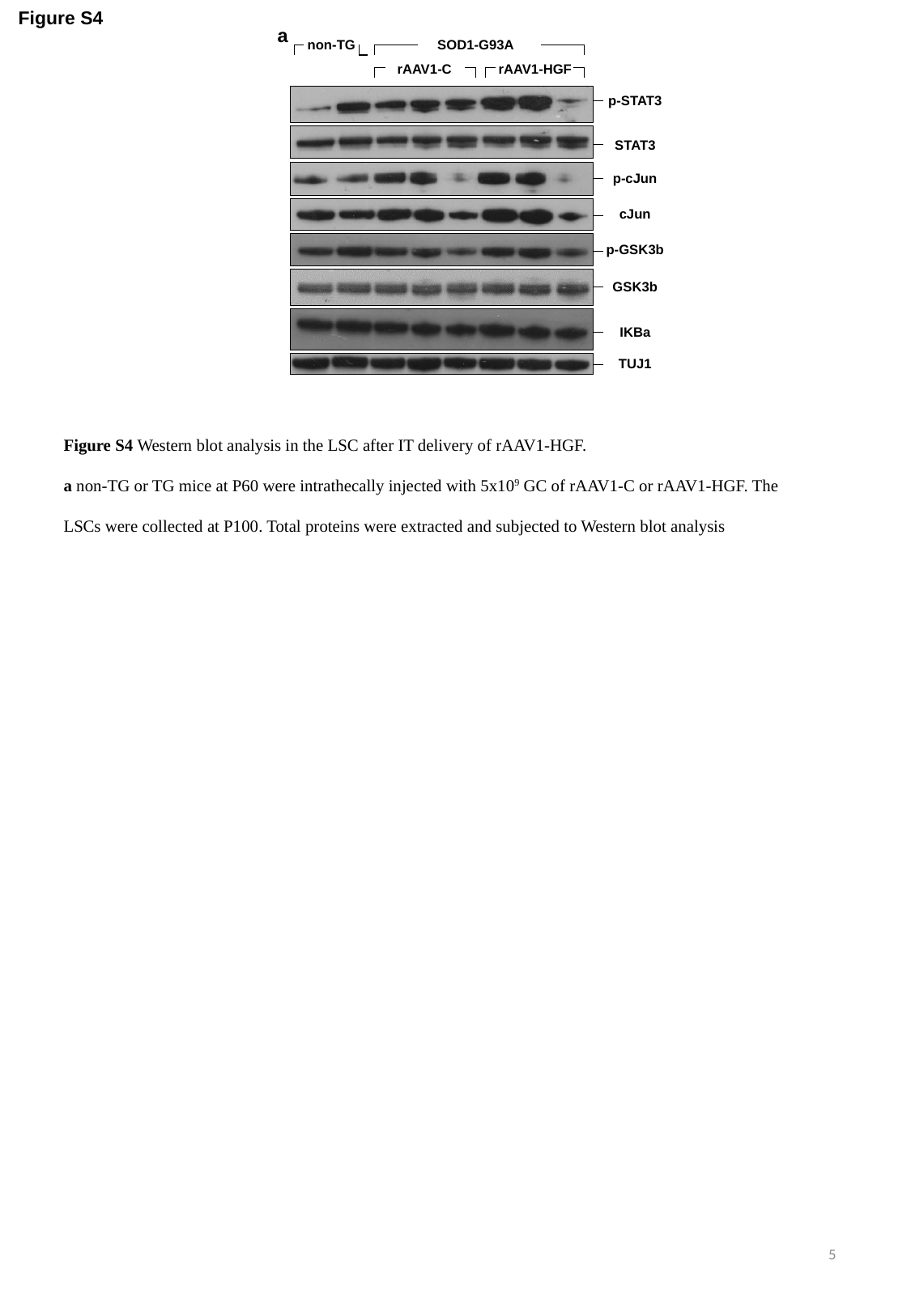

Figure S4
a
non-TG
SOD1-G93A
rAAV1-C
rAAV1-HGF
p-STAT3
STAT3
p-cJun
cJun
p-GSK3b
GSK3b
IKBa
TUJ1
Figure S4 Western blot analysis in the LSC after IT delivery of rAAV1-HGF.
a non-TG or TG mice at P60 were intrathecally injected with 5x10^9^ GC of rAAV1-C or rAAV1-HGF. The LSCs were collected at P100. Total proteins were extracted and subjected to Western blot analysis
5

## Slide 6
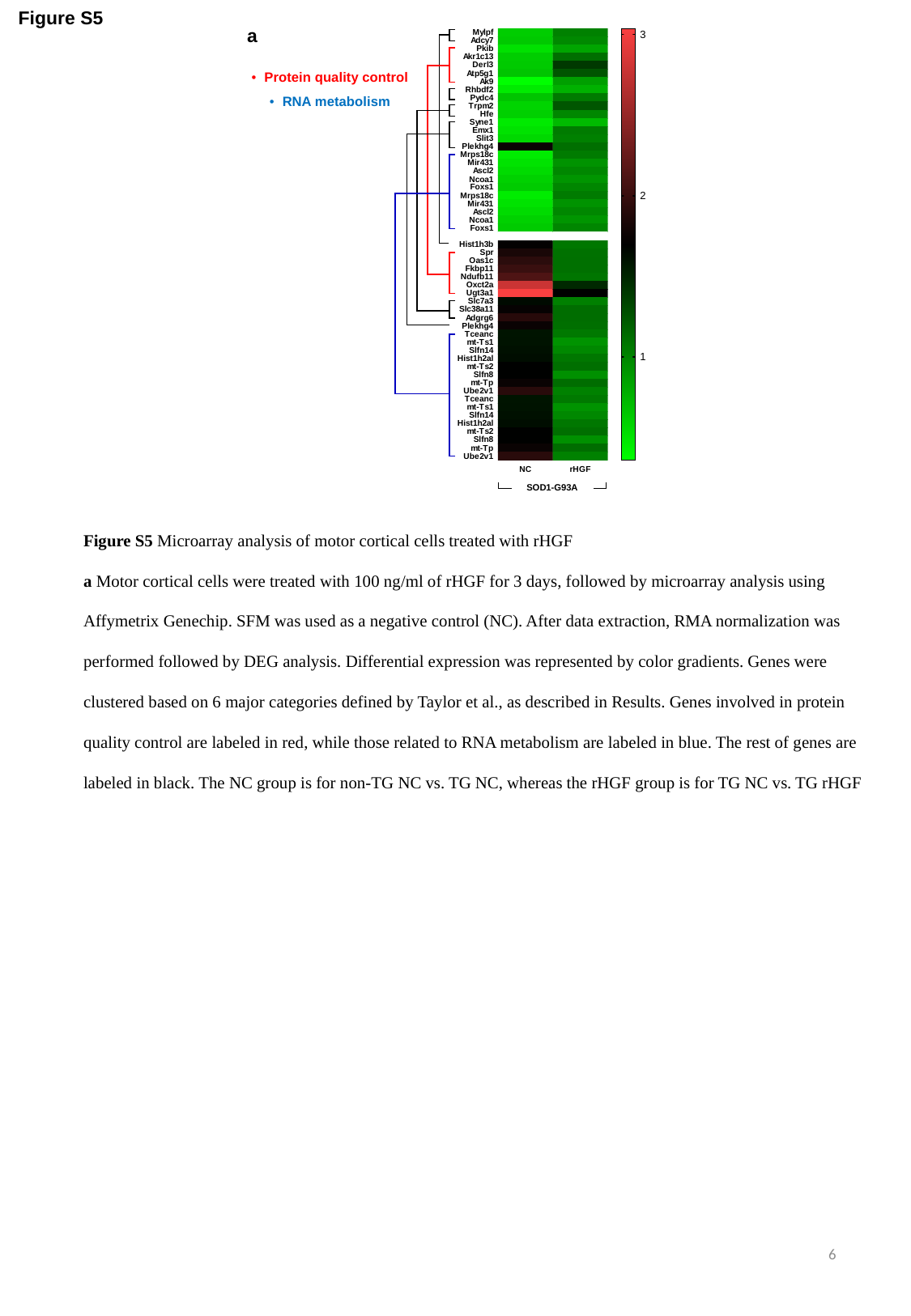

Figure S5
a
Protein quality control
RNA metabolism
Figure S5 Microarray analysis of motor cortical cells treated with rHGF
a Motor cortical cells were treated with 100 ng/ml of rHGF for 3 days, followed by microarray analysis using Affymetrix Genechip. SFM was used as a negative control (NC). After data extraction, RMA normalization was performed followed by DEG analysis. Differential expression was represented by color gradients. Genes were clustered based on 6 major categories defined by Taylor et al., as described in Results. Genes involved in protein quality control are labeled in red, while those related to RNA metabolism are labeled in blue. The rest of genes are labeled in black. The NC group is for non-TG NC vs. TG NC, whereas the rHGF group is for TG NC vs. TG rHGF
6

## Slide 7
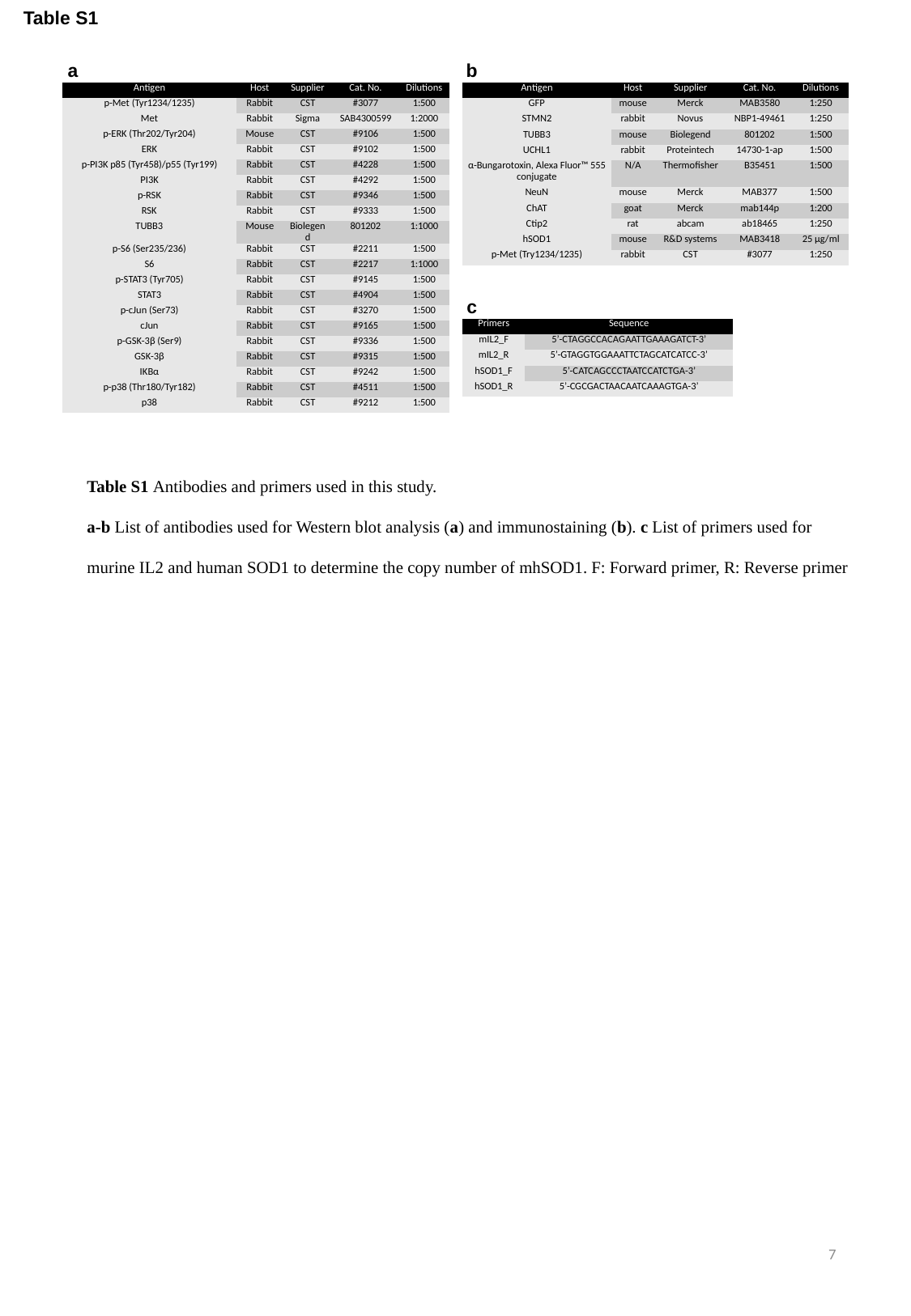

Table S1
a
b
| Antigen | Host | Supplier | Cat. No. | Dilutions |
| --- | --- | --- | --- | --- |
| p-Met (Tyr1234/1235) | Rabbit | CST | #3077 | 1:500 |
| Met | Rabbit | Sigma | SAB4300599 | 1:2000 |
| p-ERK (Thr202/Tyr204) | Mouse | CST | #9106 | 1:500 |
| ERK | Rabbit | CST | #9102 | 1:500 |
| p-PI3K p85 (Tyr458)/p55 (Tyr199) | Rabbit | CST | #4228 | 1:500 |
| PI3K | Rabbit | CST | #4292 | 1:500 |
| p-RSK | Rabbit | CST | #9346 | 1:500 |
| RSK | Rabbit | CST | #9333 | 1:500 |
| TUBB3 | Mouse | Biolegend | 801202 | 1:1000 |
| p-S6 (Ser235/236) | Rabbit | CST | #2211 | 1:500 |
| S6 | Rabbit | CST | #2217 | 1:1000 |
| p-STAT3 (Tyr705) | Rabbit | CST | #9145 | 1:500 |
| STAT3 | Rabbit | CST | #4904 | 1:500 |
| p-cJun (Ser73) | Rabbit | CST | #3270 | 1:500 |
| cJun | Rabbit | CST | #9165 | 1:500 |
| p-GSK-3β (Ser9) | Rabbit | CST | #9336 | 1:500 |
| GSK-3β | Rabbit | CST | #9315 | 1:500 |
| IKBα | Rabbit | CST | #9242 | 1:500 |
| p-p38 (Thr180/Tyr182) | Rabbit | CST | #4511 | 1:500 |
| p38 | Rabbit | CST | #9212 | 1:500 |
| Antigen | Host | Supplier | Cat. No. | Dilutions |
| --- | --- | --- | --- | --- |
| GFP | mouse | Merck | MAB3580 | 1:250 |
| STMN2 | rabbit | Novus | NBP1-49461 | 1:250 |
| TUBB3 | mouse | Biolegend | 801202 | 1:500 |
| UCHL1 | rabbit | Proteintech | 14730-1-ap | 1:500 |
| α-Bungarotoxin, Alexa Fluor™ 555 conjugate | N/A | Thermofisher | B35451 | 1:500 |
| NeuN | mouse | Merck | MAB377 | 1:500 |
| ChAT | goat | Merck | mab144p | 1:200 |
| Ctip2 | rat | abcam | ab18465 | 1:250 |
| hSOD1 | mouse | R&D systems | MAB3418 | 25 µg/ml |
| p-Met (Try1234/1235) | rabbit | CST | #3077 | 1:250 |
c
| Primers | Sequence |
| --- | --- |
| mIL2\_F | 5’-CTAGGCCACAGAATTGAAAGATCT-3’ |
| mIL2\_R | 5’-GTAGGTGGAAATTCTAGCATCATCC-3’ |
| hSOD1\_F | 5’-CATCAGCCCTAATCCATCTGA-3’ |
| hSOD1\_R | 5’-CGCGACTAACAATCAAAGTGA-3’ |
Table S1 Antibodies and primers used in this study.
a-b List of antibodies used for Western blot analysis (a) and immunostaining (b). c List of primers used for murine IL2 and human SOD1 to determine the copy number of mhSOD1. F: Forward primer, R: Reverse primer
7
